# Gram-scale synthesis of coordination polymer nanodots with renal clearance properties for cancer theranostic applications

**DOI:** 10.1038/ncomms9003

**Published:** 2015-08-06

**Authors:** Fuyao Liu, Xiuxia He, Hongda Chen, Junping Zhang, Huimao Zhang, Zhenxin Wang

**Affiliations:** 1 State Key Laboratory of Electroanalytical Chemistry, Changchun Institute of Applied Chemistry, Chinese Academy of Sciences, Changchun 130022, China; 2 University of Chinese Academy of Sciences, Beijing 100039, China; 3 Department of Biological Engineering, School of Life Science and Technology, Changchun University of Science and Technology, Changchun 130022, China; 4 Department of Radiology, The First Hospital of Jilin University, Changchun 130021, China

## Abstract

An ultrasmall hydrodynamic diameter is a critical factor for the renal clearance of nanoparticles from the body within a reasonable timescale. However, the integration of diagnostic and therapeutic components into a single ultrasmall nanoparticle remains challenging. In this study, pH-activated nanodots (termed Fe-CPNDs) composed of coordination polymers were synthesized via a simple and scalable method based on coordination reactions among Fe^3+^, gallic acid and poly(vinylpyrrolidone) at ambient conditions. The Fe-CPNDs exhibited ultrasmall (5.3 nm) hydrodynamic diameters and electrically neutral surfaces. The Fe-CPNDs also exhibited pH-activatable magnetic resonance imaging contrast and outstanding photothermal performance. The features of Fe-CPNDs greatly increased the tumour-imaging sensitivity and facilitated renal clearance after injection in animal models *in vivo*. Magnetic resonance imaging-guided photothermal therapy using Fe-CPNDs completely suppressed tumour growth. These findings demonstrate that Fe-CPNDs constitute a new class of renal clearable nanomedicine for photothermal therapy and molecular imaging.

## Abstract (web summary)

Coordination polymers are promising drug delivery nanomaterials as their structural properties can be easily controlled. Here, Wang *et al*. prepare coordination nanodots that integrate cancer imaging and therapeutic functions with beneficial renal clearance.

## Graphical abstract

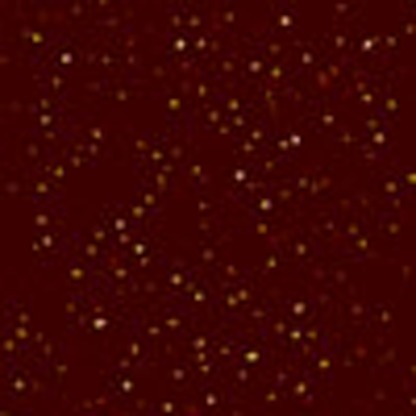

Recent developments in the field of theranostic nanomedicine have extended our ability to improve the quality of life and survival rate of patients by providing personalized medicine^1^,^2^,^3^,^4^,^5^. Because of their tuneable physicochemical properties, various inorganic/organic nanoparticles (NPs), including carbon nanotubes, metallic NPs and liposomes, have been synthesized and employed to fabricate nanomedicines for disease diagnosis and drug delivery^6^,^7^,^8^,^9^. In particular, coordination polymer-based NPs (also known as nano-sized metal–organic coordination polymers or nanoscale metal–organic frameworks) are considered a promising form of nanomedicine because they display the attractive advantages of both inorganic and organic NPs, such as a relatively regular shape, hard texture, compositional diversity, biodegradability and ease of surface functionalization^10^,^11^,^12^. For example, Lin and co-authors reported the self-assembly of a zinc bisphosphonate nanoscale coordination polymers platform designed for the selective delivery of cisplatin and oxaliplatin to tumours^10^.

Most NPs suffer from the problem of high uptake in reticuloendothelial system organs (for example, the liver and spleen), resulting in their slow and inefficient elimination via hepatobiliary excretion^13^,^14^,^15^. However, according to the US Food and Drug Administration guidelines, any imaging agent (administered into the body) should be completely cleared from the body within a reasonable period of time^16^. The long residence time of NPs in the body increases the likelihood of their toxicity. Directly excreting NPs from kidneys to the bladder can solve this problem because renal clearance is faster than hepatic clearance^16^,^17^,^18^,^19^,^20^. Because of the unique structure of the glomerular capillary wall, only NPs with ultrasmall hydrodynamic diameters can pass through the kidney^17^,^19^,^20^. Both highly anionic and cationic NPs tend to adsorb serum proteins and other species in the blood, which significantly increases their hydrodynamic diameter^16^,^18^. Therefore, fabricating renal clearable coordination polymer NPs with desirable multiple functionalities remains challenging. Nevertheless, renal clearance is a double-edged sword in clinical applications: the side effects and toxicity of NPs need to be reduced, but reducing the retention time of NPs in tumours weakens the molecular imaging sensitivity and therapeutic effects. Thus, NPs must satisfy the balance between the imaging and therapeutic requirements and the need for renal clearance within a reasonable timescale^16^,^19^,^21^. For instance, conducting a magnetic resonance imaging (MRI) within 2–4 h of the administration of NPs can satisfy the basic requirements of clinical diagnosis^22^.

Several smart NP-based systems that maximize the signal from the target and minimize the background have been designed to respond to specific biological stimuli^23^,^24^,^25^,^26^,^27^. The pH environment of tumour tissue is one of the most promising stimuli for exploring this concept because acidosis is a striking feature and universal phenomenon of solid tumours^28^. For example, Gao and co-authors established a series of ultra pH-sensitive nanoprobes to specifically image the tumour extracellular milieu and angiogenic tumour vessels^27^. Consequently, introducing ultrasmall NPs with theranostic functionalities that can be activated by pH would be of great value because these NPs provide more flexibility for multiplexing in biomedical applications, such as diagnosis and therapy.

In this study, ultrasmall poly(vinylpyrrolidone) (PVP)-protected Fe^3+^-gallic acid (GA) coordination polymer nanodots (Fe-CPNDs) that can be activated by the pH are synthesized via a simple and scalable strategy using non-hazardous materials. The hydrodynamic diameter of Fe-CPNDs is small (5.3 nm), their surface is electrically neutral, and they display excellent photothermal properties and high colloidal stability. *In vivo* animal experiments demonstrate that the Fe-CPNDs accumulated in tumour sites via the enhanced permeability and retention (EPR) effect and are completely excreted by renal clearance 24 h after tail-vein injection. These features comply with standard requirements for clinical applications. Moreover, the MRI contrast of Fe-CPNDs, which is activated by the pH, is excellent, and they effectively ablate tumours *in vivo*, which highlights the clinical application potential of Fe-CPNDs as theranostic probes for imaging-guided cancer therapy.

## Results

### Synthesis and characterization of Fe-CPNDs

**Figure 1:**
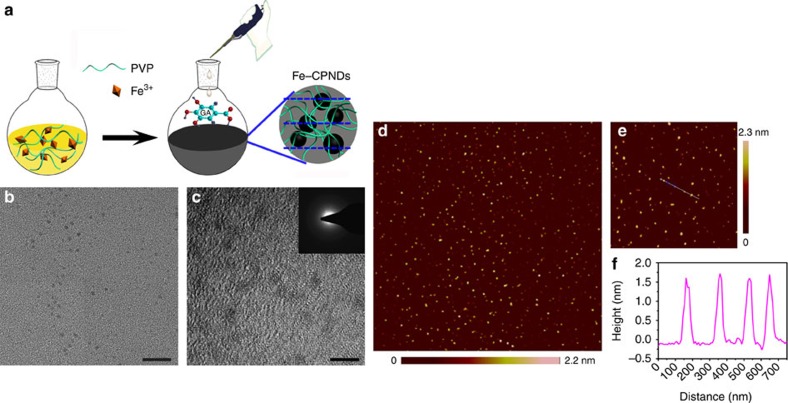
Synthesis and characterization of Fe-CPNDs. (**a**) Schematic illustration of the synthesis of Fe-CPNDs. (**b**) TEM micrograph (scale bar, 20 nm) and (**c**) high-resolution TEM micrograph of Fe-CPNDs (scale bar, 5 nm). (**c**, inset) The corresponding FFT pattern of Fe-CPNDs. (**d**,**e**) AFM topography images of Fe-CPNDs. (**f**) The height profile along the line marked in the AFM image (**e**).

We report a simple and robust method for preparing nanodots of controllable morphology and size based on a one-step assembly of phenolic group–metal ion coordination complexes. The principle of this method is illustrated in Fig. 1a. GA and Fe^3+^ were selected as the organic ligand and inorganic cross-linkers, respectively. Fe^3+^ is less biotoxic than other paramagnetic ions (for example, Gd^3+^ and Mn^2+^). GA is a type of low-molecular-weight tea polyphenol that can react with Fe^3+^ to form a stable GA_*n*_-Fe^3+^ (*n*≤3) complex via the formation of phenolate carboxylate group–Fe^3+^ coordination bonds. PVP–Fe^3+^ complexes are immediately formed on the mixing of PVP and Fe^3+^ in water at ambient temperatures^29^,^30^. The introduction of GA to the aqueous dispersion of PVP–Fe^3+^ complexes allows the formation of coordination polymer nanodots at the reactive site of the complexes at which GA contacts the Fe^3+^. PVP also serves as a protecting polymer during the nucleation and growth processes of coordination polymer nanodots because the amide moieties of PVP are weekly coordinated to Fe^3+^ ions. Therefore, PVP can sterically stabilize the nanodots. The weak interaction between PVP and Fe^3+^ was confirmed using X-ray photoelectron spectroscopy (XPS), infrared spectroscopy and Raman spectroscopy. After being coordinated with Fe^3+^, the peak of the amide group in the O 1*s* spectra shifted from 530.5 to 531.4 eV (Supplementary Fig. 1; Supplementary Methods), which suggests an interaction between PVP and Fe^3+^. The Fourier transform infrared (FTIR) spectrum of PVP–Fe^3+^ shows a band at 1,670 cm^−1^ (C=O stretching of the PVP amide unit) and a shoulder band at ∼1,600 cm^−1^ (Supplementary Fig. 2). The appearance of the latter shoulder peak suggests that part of the amide unit in PVP binds to the Fe^3+^ ions^29^. A new band at 1,600 cm^−1^ was clearly observed in the Raman spectrum of PVP–Fe^3+^, further demonstrating the interactions of PVP with Fe^3+^ ions (Supplementary Fig. 3). As expected, monodispersed coordination polymer nanodots (Fe-CPNDs) were successfully prepared (Fig. 1b,c). The X-ray absorption fine structure analysis suggests that the Fe element mainly exists in the ferric form in the Fe-CPNDs, and one iron atom coordinates with six oxygen atoms (Supplementary Figs 4–7; Supplementary Table 1; Supplementary Discussion; Supplementary Methods). Few well-shaped particles were observed in the absence of PVP, Fe^3+^ or GA (Supplementary Fig. 8; Supplementary Methods). This result demonstrates that the method enables the synthesis of Fe-CPNDs at ambient conditions using simple equipment. In particular, the material components are readily available, inexpensive and generally recognized as safe by the US Food and Drug Administration. For instance, GA is a phytochemical commonly used in the pharmaceutical industry^31^,^32^,^33^. PVP has been used as an ideal stabilizer for NP-based contrast agents in various medical applications because of its nontoxicity, neutrality, long blood circulation times and low accumulation in vital organs^34^. In particular, Fe-CPNDs can be easily achieved produced on a large scale by increasing the amount of reactants, and the as-prepared hydrodynamic diameter of Fe-CPNDs remains unchanged after multiple drying-dissolving processes (Supplementary Fig. 9; Supplementary Methods). The results reveal that this method may be advantageous for industrialization.

The as-prepared Fe-CPNDs are spherical particles with an average diameter of 1.49±0.21 nm and a relatively narrow size distribution (Fig. 1b,c; Supplementary Fig. 10). Fe-CPNDs show low diffraction contrast without obvious lattice fringes in the high-resolution transmission electron microscopy (TEM) measurements, indicating that the nanodots are amorphous (as shown in inset of Fig. 1c). Furthermore, the size and morphology of the Fe-CPNDs were characterized by atomic force microscopy (AFM, Fig. 1d–f). The Fe-CPNDs are spherical NPs with an average size of 1.51±0.19 nm. The hydrodynamic diameter of Fe-CPNDs is small, ∼5.3 nm, in both the PBS buffer and serum, ensuring that the Fe-CPNDs satisfy the size threshold requirements for renal clearance (Supplementary Fig. 11)^16^,^17^,^18^,^19^,^20^. The hydrodynamic diameter of the NP exceeds the diameter of the NP as determined by TEM under a high vacuum. Therefore, the outer PVP layer significantly increases the hydrodynamic diameter of the Fe-CPNDs. Considering that the hydrodynamic diameter of PVP (molecular weight=8,000) is ∼2 nm in water, the observed dynamic light scattering (DLS) data confirm that the Fe-CPNDs are coated with random coils of PVP chains. The hydrodynamic diameters and zeta potentials of the Fe-CPNDs in different dispersants are shown in Supplementary Table 2. The Fe-CPNDs display a good monodispersity in all cases. The nearly neutral zeta potential (−3.76 mV) of Fe-CPNDs suggests that the surfaces of the Fe-CPNDs are coated with the corresponding nonionic hydrophilic PVP polymers (Supplementary Fig. 12). This PVP coating stabilizes the Fe-CPNDs in different dispersants for several months, including H_2_O, PBS, TB, 0.9% NaCl solution and DMEM supplemented with 10% fetal bovine serum (v/v), (Supplementary Fig. 13). The presence of Fe in the Fe-CPNDs was also confirmed by energy-dispersive X-ray spectroscopy (Supplementary Fig. 14) and XPS (Supplementary Fig. 15) analyses. The X-ray diffraction data further confirm that the Fe-CPNDs are amorphous materials (Supplementary Fig. 16). The infrared intensity of Fe-CPNDs at 1,250 cm^−1^ (the HO–C stretching band) is lower than that of GA, indicating that the HO–C moiety of GA coordinates with Fe^3+^. The infrared bands at 2,955 cm^−1^ (C–H stretching) and 1,654 cm^−1^ (C=O) in the FTIR spectrum of the Fe-CPNDs (Supplementary Fig. 17) further confirm the presence of PVP on the nanodots^35^.

### MRI of Fe-CPNDs

**Figure 2:**
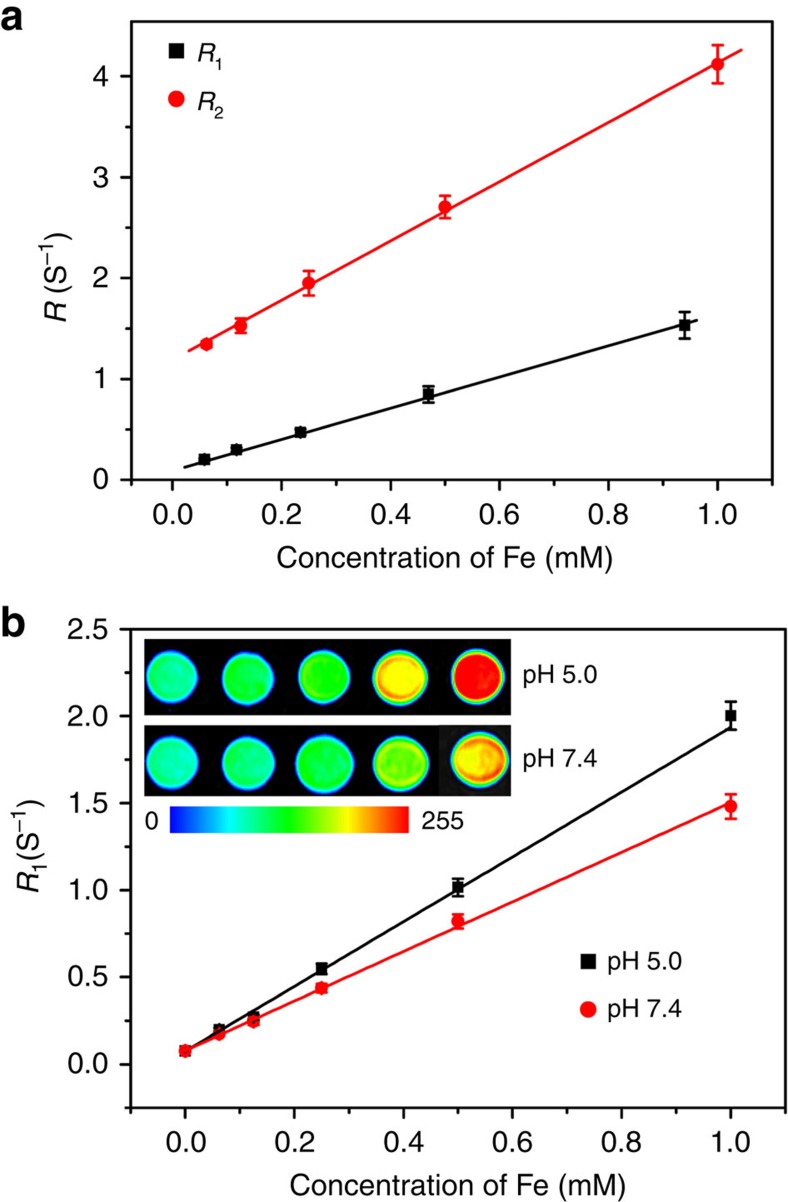
Magnetic resonance contrast effect of Fe-CPNDs. (**a**) *R*_1_ and *R*_2_ relaxivities of Fe-CPNDs as a function of the molar concentration of Fe^3+^ in the solution. (**b**) *R*_1_ relaxivity of Fe-CPNDs as a function of the molar concentration of Fe^3+^ in the solution for different pH values. (**b**, inset) Corresponding MRI images of Fe-CPNDs under different pH values.

The longitudinal (*r*_1_) and transverse (*r*_2_) relaxivities were measured to examine the feasibility of using Fe-CPNDs as *T*_1_ MRI contrast agents (Fig. 2a). The *r*_1_ and *r*_2_ relaxivities of the Fe-CPNDs are 1.5 and 2.9 mM^−1^ s^−1^, respectively, which are comparable to those of reported contrast agents^36^,^37^,^38^. The values of *r*_1_ and *r*_2_/*r*_1_ (ca. 1.9) suggest that the Fe-CPNDs can be used as highly efficient *T*_1_ contrast agents^39^. The coordination number of TA (tannic acid) in Fe^3+^–TA_*n*_ complexes is increased by increasing the solution pH value (that is, the Fe^3+^–TA, Fe^3+^–TA_2_ and Fe^3+^–TA_3_ complexes dominate at pH <2, pH=3–6 and pH >7, respectively)^40^. Because GA is the monomer of TA, GA may also form pH-dependent coordination complexes. For example, three GA molecules can react with one Fe^3+^ to form a stable complex (Fe^3+^–GA_3_) under physiological conditions. However, Fe^3+^–GA_3_ will gradually dissociate in mildly acidic environments, and Fe^3+^–GA_*n*_ complexes consequently transform from tris-coordination to bis-coordination. The pH value similarly affects the ultraviolet−visible spectra of the as-prepared Fe-CPNDs and Fe^3+^–TA_*n*_ complexes (Supplementary Fig. 18)^40^. The spectral changes of Fe-CPNDs can be attributed to transitions between the mono-, bis- and tris-complex states of Fe^3+^–GA_*n*_ complexes. The reduced coordination number in mildly acidic conditions could enhance the quantity and the mobility of the coordinated water in the first and second coordination spheres surrounding the Fe paramagnetic centre to further improve the relaxivity. Infrared spectra at different temperatures confirmed the coordinated water in degraded Fe-CPNDs (Supplementary Fig. 19; Supplementary Methods). Furthermore, more CO adsorbed to the Fe-CPNDs at pH 5.0 than at pH 7.4, indicating that the Fe-CPNDs contain more activated Fe^3+^ sites at pH 5.0 (Supplementary Fig. 20; Supplementary Methods). As expected, we observed a significant increase in the *r*_1_ value, from 1.4 to 1.9 mM s^−1^ (Fig. 2b), when the pH was adjusted from 7.4 to 5.0. The MRI contrast, which is activated by the pH value, is beneficial to tumour detection. For instance, the performance of Fe-CPNDs during MRI could greatly improve at the tumour site because tumour tissues are normally more acidic than normal tissues. After incubation at 37 °C for a given period, the absorbance of Fe-CPNDs decreased, and the colour of the solution gradually faded as the solution pH decreased. The results suggest that Fe-CPNDs can degrade (Supplementary Fig. 21). The degradation products were further analysed based on the high-performance liquid chromatography, fast Fourier transfer (FFT) patterns and XPS. The amount of free GA in the solution positively correlated with the incubation time and negatively correlated with the pH value (Supplementary Fig. 22; Supplementary Methods). The XPS spectrum and FFT patterns of Fe-CPNDs display negligible changes after degradation (Supplementary Figs 23 and 24). The results demonstrate that GA molecules gradually dissociated from Fe-CPNDs in mildly acidic environments, and the dissociation of GA did not generate a new crystalline phase.

To evaluate the MRI imaging capability, *T*_1_-weighted magnetic resonance images of the Fe-CPND-stained SW620 cell pellets were gathered on a Siemens 1.5 T MRI scanner. The magnetic resonance signal intensity positively correlated with the concentration of Fe-CPNDs in the culturing medium. These experimental results highlight the labelling capability of CPNDs and demonstrate their potential application as molecular MRI agents (Supplementary Fig. 25).

**Figure 3:**
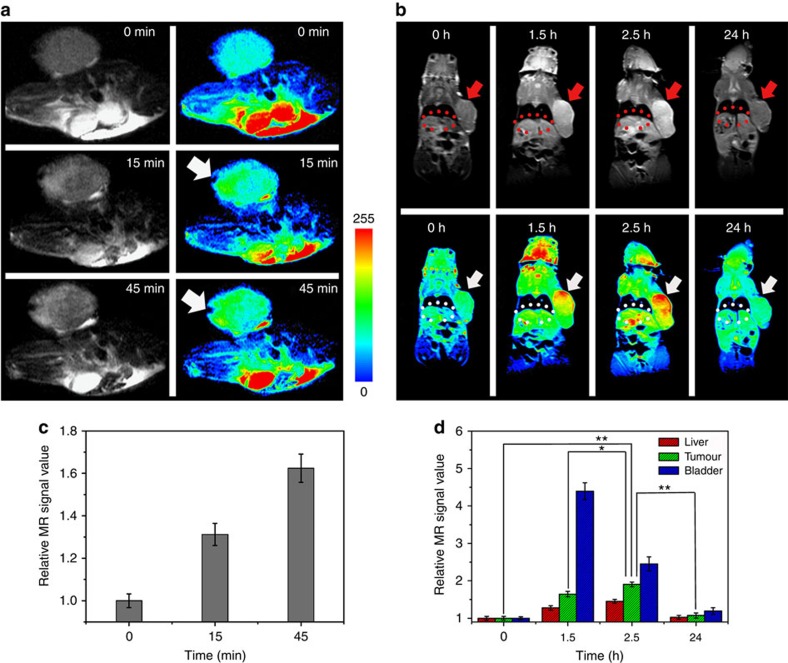
*In vivo* MRI of colorectal tumour xenografts. *In vivo* magnetic resonance images of nude mice bearing colorectal tumours after (**a**) intratumour injection (injection site, arrows) and (**b**) intravenous injection (tumour, arrows; liver, ellipses) of Fe-CPNDs at different time intervals (0 min and 0 h indicate pre-injection). (**c**) Corresponding data analysis of the tumour in **a**. (**d**) Corresponding data analysis of the tumour, bladder and liver in **b**. Error bars indicate the s.d. (*n*=5, **P<0.01 or *P<0.05 from an analysis of variance with Tukey's post-test). The tumours are ∼700 mm^3^ in volume.

Fe-CPNDs provide an excellent platform to enhance the MRI performance using the pH because of the pH-dependent coordination reaction between Fe^3+^ and GA. To examine the suitability of this pH-sensitive probe for *in vivo* tumour imaging, a nude mouse colorectal (SW620) tumour model was established for an *in vivo* MRI evaluation. The MRI studies show that the contrast of the *T*_1_ MRI image was enhanced 15 min after the intratumour administration of NPs (Fig. 3a; Supplementary Fig. 26). Accompanying the acid-induced Fe^3+^–GA_*n*_ complex transition in the tumour region, the *T*_1_-weighted magnetic resonance image brightened over time (Fig. 3a,c). Correspondingly, the signal enhancement area gradually expanded from the injection site to the entire tumour (Fig. 3a) because of the diffusion process of Fe-CPNDs. This phenomenon indicates that these Fe-CPNDs could be utilized for ultrasensitive MRI applications because of their ability to be activated by the pH. For comparison, Fe-CPNDs were also injected into the thigh muscles of a nude mouse. The magnetic resonance signal of the thigh muscles remained unchanged over time (Supplementary Fig. 27). These results further demonstrate the acidic environment in the tumour can activate the Fe-CPNDs.

**Figure 4:**
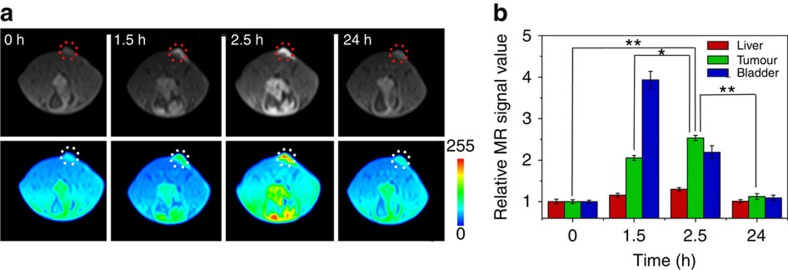
*In vivo* MRI of very small colorectal tumour xenografts. (**a**) *In vivo* magnetic resonance images of the selected nude mice bearing very small (∼5 mm^3^ in volume) colorectal tumours (tumour, circles) after the intravenous injection of Fe-CPNDs at different time intervals (0 h indicates pre-injection). (**b**) Corresponding data analysis of the tumour and main organs in **a**. Error bars indicate the s.d. (*n*=5, **P<0.01 or *P<0.05 from an analysis of variance with a Tukey's post-test). Each nude mouse was scanned five times to calculate s.d.

Building on these results, we evaluated the potential preferential tumour accumulation of the NPs in a subcutaneous SW620 cancer model using a Siemens 1.5 T MRI scanner. Whole-body MRI images were obtained 1.5, 2.5 and 24 h after the intravenous injection of Fe-CPNDs. A sustained increase of the *T*_1_ contrast in the tumour site was observed from 0 to 2.5 h post Fe-CPND injection, indicating the effective uptake of Fe-CPNDs in the tumour via the EPR effect (Fig. 3b; Supplementary Fig. 28). The maximum MRI signal enhancement of the tumour site was achieved 2.5 h post injection. At this time, the proximal edge of the tumour was effectively delineated by the MRI (Fig. 3b). Notably, the signal change was small in the liver over this period (Fig. 3b,d). The high accumulation of Fe-CPNDs in the tumour could be attributed to their size and structural features. The ultrasmall hydrodynamic diameter (5.3 nm) of Fe-CPNDs benefits the EPR and nanomaterial-induced endothelial cell leakiness (NanoEL) effects. In addition, the pH-activated MRI contrast of Fe-CPNDs assists the detection of the tumour microenvironment^22^,^27^,^41^,^42^,^43^. The signal enhancement in the bladder is maximized 1.5 h post injection. At the final time point (24 h after intravenous injection), a negligible signal enhancement in the tumour and organs (for example, the liver and bladder) was observed (Fig. 3b,d), indicating the excretion of Fe-CPNDs from the circulation. To further evaluate the tumour-targeting ability of Fe-CPNDs, Fe-CPNDs were injected into nude mice bearing small tumour xenografts (5 mm^3^ in volume) through the tail vein. The MRI signal-enhancing process of the tumour site is consistent with the experimental results of a large tumour (Fig. 4; Supplementary Fig. 29). These experimental results demonstrate that the as-prepared Fe-CPNDs can be efficiently enriched in both large and small tumours via the EPR.

The amounts of Fe in the main organs were measured using inductively coupled plasma mass spectroscopy (ICP-MS; Supplementary Figs 30 and 31; Supplementary Methods). For intravenous injection, the kidney and tumour accumulation dominate the biodistribution of the Fe-CPNDs throughout the treatment. The amount of Fe in the tumour was maximized 2.5 h after injection, and the amount of Fe began to decrease between 2.5 and 24 h after injection. The result is consistent with the MRI study. For intratumour injection, the Fe-CPNDs had primarily accumulated in the tumour 45 min after injection. This phenomenon is also consistent with the previous MRI study.

In addition, the effects of the Fe-CPND concentration on the MRI signal were investigated (Supplementary Figs 32 and 33). The MRI signal was enhanced at Fe-CPND concentrations as low as 0.8 mg kg^−1^. Therefore, we estimate that the minimum dose required to significantly enhance the MRI signal is 0.8 mg kg^−1^.

### PTT of Fe-CPNDs

**Figure 5:**
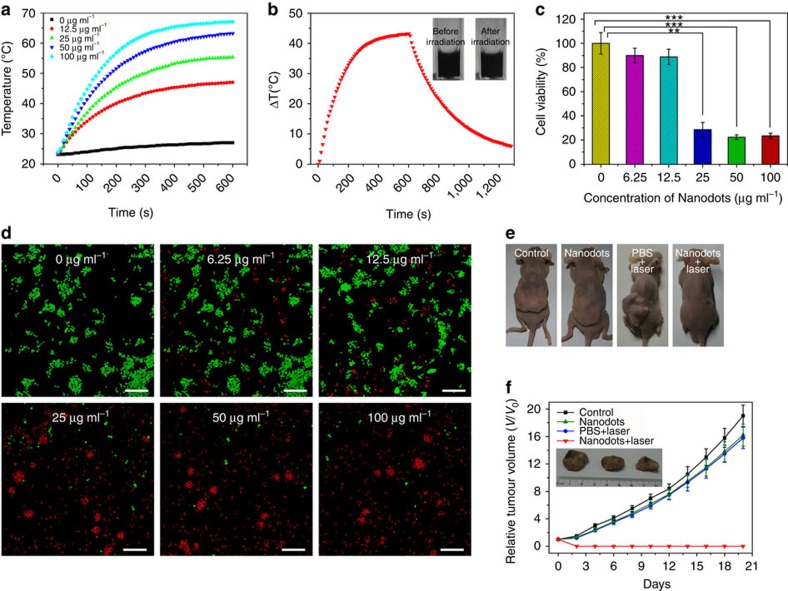
Photothermal therapy of Fe-CPNDs. (**a**) Temperature elevation of the water and Fe-CPNDs solutions of various concentrations (Fe content: 0–100 μg ml^−1^) over a 10-min exposure to 808-nm NIR light (1.3 W cm^−2^). The temperatures were measured every 10 s using a thermometer. (**b**) The photothermal response of the 100 μg ml^−1^ (Fe content) Fe-CPND aqueous solution when exposed to 808-nm NIR light (1.3 W cm^−2^) for 600 s. The laser was then turned off. The inset shows the digital photograph of the dispersion of Fe-CPNDs in water before and after laser irradiation. (**c**) *In vitro* cell viabilities of SW620 cells incubated with various concentrations of Fe-CPNDs (Fe content: 0–100 μg ml^−1^) under 808-nm NIR laser irradiation (1.3 W cm^−2^, 6 min). The error bars indicate the s.d. (*n*=5, **P<0.01 or ***P<0.001 from an analysis of variance with Tukey's post-test). (**d**) Confocal fluorescence images of SW620 cells after incubation with various concentrations of Fe-CPNDs (Fe content: 0–100 μg ml^−1^) under 808-nm NIR laser irradiation for 6 min. The cells were co-stained with calcein acetoxymethyl ester (green, living cells) and propidium iodide (red, dead cells). Scale bar, 200 μm. (**e**) Digital photographs of the mice collected from different groups at the end of intravenous treatments (day 20). (**f**) Corresponding tumour growth curves of different groups of mice after intravenous treatments. Error bars indicate the s.d. (*n*=4). The inset shows the digital photographs of tumours collected from different groups of mice at the end of intravenous treatments (day 20).

The Fe-CPNDs are bluish black because of a strong ligand-to-metal charge transfer band from the phenolic oxygens^44^. Therefore, Fe-CPNDs strongly absorb light in the visible to near-infrared (NIR) region (Supplementary Fig. 34), which motivated us to investigate their photothermal effects. Figure 5a shows the heating curve of the Fe-CPND solutions of varying concentrations using an 808-nm diode laser at 1.3 W cm^−2^ irradiation density. As expected, the temperatures of Fe-CPNDs aqueous solutions rapidly increased when he NIR irradiation time was increased from 0 to 5 min and begins to plateau after 5 min (Fig. 5a). The increase in the temperature and the final solution temperature are proportional to the concentration of Fe-CPNDs. In the presence of 25 μg ml^−1^ Fe-CPNDs (Fe content), the solution temperature successfully increased to 50 °C (a temperature that efficiently kills cancerous cells)^45^ after 5 min of NIR irradiation. Furthermore, the absorption of Fe-CPNDs irradiated 808-nm NIR laser for over 60 min did not significantly change (Supplementary Fig. 35), thus indicating the high photostability of Fe-CPNDs. These results suggest that Fe-CPNDs are suitable agents for use in the photothermal therapy (PTT) of cancers.

3-(4,5-dimethylthiazol-2-yl)-2,5-diphenyltetrazolium bromide (MTT) assays of different cells were performed to test the cytotoxicity of Fe-CPNDs. After being treated Fe-CPND concentrations up to 200 μg ml^−1^, both normal cells (HL-7702 and MC3T3-E1) and tumour cells (SW620 and HepG2) showed >90% cell viability. This result suggests that the Fe-CPNDs are minimally cytotoxic at a wide range of concentrations (Supplementary Fig. 36). These results are reasonable because all three components of the Fe-CPNDs (GA, Fe^3+^ and PVP) have previously been shown to display low cytotoxicity^39^,^46^,^47^. Typically, the MTT assay is used to evaluate the PTT efficacy of Fe-CPNDs in SW620 cells based on cell viability. On 808-nm NIR laser irradiation, the Fe-CPNDs are highly cytotoxic to SW620 cells in a dose-dependent manner (Fig. 5c). To further identify the PTT efficacy of Fe-CPNDs *in vitro*, 808-nm NIR-irradiated and non-irradiated Fe-CPND-treated SW620 cells were co-stained with calcein acetoxymethyl ester and propidium iodide and imaged by confocal microscopy (Fig. 5d). The confocal microscopy result is consistent with the results of the MTT assay.

Encouraged by the high PTT efficiency *in vitro* and the efficient passive tumour targeting of Fe-CPNDs, *in vivo* PTT studies were performed to evaluate the anticancer efficacy of Fe-CPNDs in a xenograft tumour mouse model (Fig. 5e,f; Supplementary Fig. 37). For the PTT group, the mice were intravenously injected with various concentrations of Fe-CPNDs and then irradiated for 6 min with the 808-nm laser at 1.3 W cm^−2^ 2.5 h after injection. The tumour sizes were measured with a calliper every 2 days. As expected, the temperature of tumour site positively correlated with the irradiation time (Supplementary Fig. 38; Supplementary Methods). PTT is efficient for an Fe-CPND concentration of 0.2 mg kg^−1^ (Supplementary Fig. 37). As illustrated in Fig. 5f, Fe-CPND-mediated PTT can significantly inhibit tumour growth, whereas the PBS plus 808-nm laser irradiation or Fe-CPNDs only treatments exhibited negligible tumour suppression. The Fe-CPND-mediated PTT-treated tumours were completely ablated on day 20, leaving black scars at the original sites (Fig. 5e). The experimental results indicate that Fe-CPNDs facilitate MRI-guided tumour PTT *in vivo*.

### *In vivo* biodistribution and toxicology investigation

**Figure 6:**
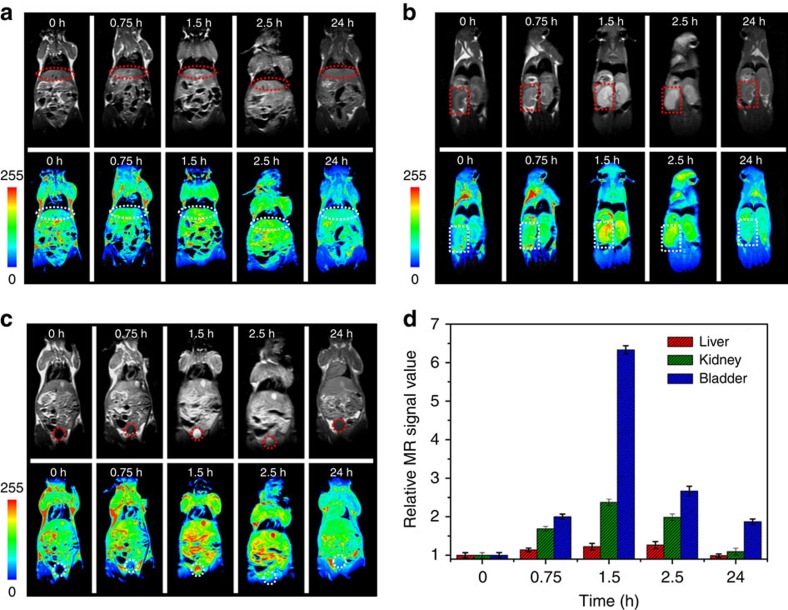
*In vivo* biodistribution and clearance of Fe-CPNDs. *In vivo* magnetic resonance images of the (**a**) liver (ellipses), (**b**) kidney (rectangles) and (**c**) bladder (circles) after the intravenous injection of Fe-CPNDs at different time intervals (0 h indicates pre-injection.). (**d**) Corresponding data analysis of the magnetic resonance measurements. Error bars indicate the s.d. (*n*=5).

The hydrodynamic diameter of Fe-CPNDs is ultrasmall, and their surface is neutral, which could help them pass through the kidney to allow these NPs to be eliminated from the body. This process decreases the biological persistence of NPs injected into the body and eliminates the risks of long-term toxicity.^14^,^15^,^16^ *In vivo* MRI was employed to investigate the biodistribution and clearance of Fe-CPNDs in normal (healthy) mice. The Fe-CPNDs were intravenously injected into the animals, and *T*_1_-weighted magnetic resonance images were acquired before and 0.75, 1.5, 2.5 and 24 h after injection. An initial signal increase was noted in the kidneys and bladder; this increase was followed by signal decay starting after 1.5 h (Fig. 6b–d; Supplementary Fig. 39). Twenty-four hours after intravenous injection, the signal in the kidneys recovered to the pre-injection levels, and the signal in the bladder also significantly decreased, indicating the excretion of the Fe-CPNDs from the body via renal filtration. In addition, the signal change in the liver was relatively small throughout the course of the experiment, indicating the low accumulation of Fe-CPNDs in the reticuloendothelial system organs (Fig. 6a,d). These results provide additional evidence of the renal clearance of the injected Fe-CPNDs. The amount of Fe in the blood of the mouse was measured at different time points after injection to evaluate the pharmacokinetics of Fe-CPNDs. The Fe-CPNDs followed a two-compartment pharmacokinetic process, with a distribution half-life (*t*_1/2α_) of 0.25±0.04 h and a blood-elimination half-life (*t*_1/2β_) of 5.5±1.9 h (Supplementary Fig. 40; Supplementary Methods). The mouse urine was collected during the treatment for ICP-MS, MRI and TEM measurements. We found that the total amount of Fe in the urine and the MRI signal of the urine increased over time (Supplementary Fig. 41; Supplementary Methods). A negligible morphology change in the Fe-CPNDs was observed by TEM (Supplementary Fig. 41). The results demonstrate that the signal reduction in mice over time was mainly due to the clearance of the NPs.

Furthermore, the *in vivo* toxicity of the Fe-CPNDs was examined with a histochemical, haematology and blood biochemical analyses. Neither death nor significant body weight loss was noted in the Fe-CPND-treated group over a course of 1 month (Supplementary Fig. 42). The major organs (for example, the cerebrum, cerebellum, heart, liver, spleen, lung, kidneys and testes) of the Fe-CPND-treated mice were collected 7 and 30 days after injection for a histology analysis. The organs of the Fe-CPND-treated mice did not exhibit noticeable abnormalities or lesions compared with those of the controls (Supplementary Fig. 43). The haematology analysis and blood biochemical assays of the Fe-CPND-treated groups are consistent with those of the controls (Supplementary Table 3), further confirming the nontoxicity of the Fe-CPNDs. The preliminary *in vivo* toxicity studies suggest that Fe-CPNDs may be used as a renal clearance theranostic nanoplatform for biomedical applications.

In summary, we demonstrated the use of the coordination assembly of Fe^3+^, GA and PVP to prepare a class of renal clearable nanodots that integrate imaging and therapeutic functions. Due to the ultrasmall hydrodynamic diameter and acidic dissociation properties of the Fe^3+^–GA_3_ complex, the as-prepared Fe-CPNDs preferentially accumulate in the tumour, are rapidly eliminated from the body via the renal system and can be activated by the low pH of the tumour to enhance MRI contrast. Fe-CPNDs also display an excellent photothermal effect, enhancing their appeal as PTT agents. Although a xenograft tumour mouse model was selected to test the utility of the nanodots, the experimental results suggest the potential of Fe-CPNDs in clinical translation as a safe and efficient theranostic nanomedicine. This potential is further strengthened by the potential industrial production of these nanodots.

## Methods

### Materials

GA (99%), iron(III) chloride hexahydrate (FeCl_3_.6H_2_O) and PVP (molecular weight=8,000 g mol^−1^) were obtained from Alfa Aesar (Ward Hill, Massachusetts, USA). Leibovitz's L-15 medium (L-15) and MTT were ordered from Sigma-Aldrich Co. (St Louis, USA). Fetal bovine serum was obtained from Gibco (New York, USA). All reagents were used without further purification. Milli-Q water (18.2 MΩ cm) was used in all experiments.

### Characterization

The TEM micrographs and FFT patterns were recorded on a TECNAI G2 high-resolution TEM (FEI Co., USA). X-ray diffraction was performed on a D8 ADVANCE diffractometer (Bruker Co., Germany) using Cu Kα (0.15406, nm) radiation. The DLS and zeta potential of the as-prepared samples were determined on a Zetasizer Nano ZS (Malvern Instruments Ltd, UK). The elemental analysis was performed on an ELAN 9000/DRC ICP-MS system (PerkinElmer, USA). The infrared spectra were captured with a Bruker Vertex 70 FTIR spectrometer. XPS measurements were conducted with a VG ESCALAB MKII spectrometer (VG Scientific Ltd, UK). The morphology and size of Fe-CPNDs were determined on an AFM using the ScanAsyst mode in air (Dimension Icon, Veeco Instruments/Bruker, Germany). Commercially available AFM cantilever tips with a force constant of ∼ 48 N m^−1^ and resonance vibration frequency of ∼330 kHz were used. The scanning rate was set to 0.4 Hz. The samples for AFM were prepared by dropping an aqueous suspension (0.2 mg ml^−1^) of Fe-CPNDs on a freshly cleaved mica surface via a spin-coating method.

### Synthesis of Fe-CPNDs

The Fe-CPNDs were synthesized at a mass ratio of PVP:Fe:GA of 66:20:10 in the starting reaction solution. Typically, 66 mg of PVP was dissolved in 8.8 ml of water at room temperature under vigorous stirring. An FeCl_3_ aqueous solution (0.2 ml, 100 mg ml^−1^) was then added to the aqueous PVP solution. After 1 h of incubation, a GA aqueous solution (1 ml, 10 mg ml^−1^) was added to the above reaction mixture and stirred overnight. The resulting nanodots were dialysed (MWCO (molecular weight cut off)=25,000) against deionized water for 24 h. On the basis of the elemental analysis (XPS quantitative analysis), the mass ratio of PVP:Fe:GA was 74.4:3.6:22 in the final Fe-CPNDs. The yield of Fe-CPNDs was ∼25%. The space-time yield was ∼2.5 mg ml^−1^ h^−1^ when 66 mg ml^−1^ PVP was used in the starting reaction solution.

In all experiments, the concentration of the Fe-CPNDs was defined by the Fe content. The mice were anaesthetized during imaging and PTT treatment using a peritoneal injection of 10% chloral hydrate (80 μl).

### MRI of phantom

To investigate the ability of the pH to activate the coordination polymer nanodots *in vitro* to enhance the MRI contrast, the Fe-CPNDs were suspended in different PBS buffer solutions (pH 7.4 and 5.0) and incubated at 37 °C for 4 h. The solutions were then diluted with a corresponding buffer solution to obtain the desired concentrations. Finally, the Fe-CPNDs solutions (1 ml) of various concentrations were transferred to 1.5 ml centrifuge tubes for the MRI test. The *T*_1_-weighted magnetic resonance images were acquired using a Siemens 1.5 T MRI scanner (Magnetom Avanto, Siemens, Erlangen, Germany) with the following imaging parameters: repetition time, 613 ms; echo time, 8.4 ms; field of view, 102 × 72 mm; and slice thickness, 3.0 mm.

### Measurement of photothermal performance

A series of aqueous solutions containing different concentrations of Fe-CPNDs (0, 12.5, 25, 50 and 100 p.p.m.) were irradiated with an 808-nm NIR laser at a power density of 1.3 W cm^−2^ for different times. The solution temperature was monitored with a thermocouple probe.

### Cell viability assay and cell uptake

All cells, including the normal (HL-7702 and MC3T3-E1) and tumour cells (SW620 and HepG2), were obtained from the Shanghai Cell Bank of the Chinese Academy of Sciences. To quantitatively evaluate the photothermal cytotoxicity of the Fe-CPNDs, SW620 cells were first incubated with the desired amounts of Fe-CPNDs. The Fe-CPND-stained cells were then irradiated with an 808-nm NIR laser (1.3 W cm^−2^) for 6 min, washed with 100 μl of fresh L-15 (three times) and cultured with 100 μl of fresh L-15 for another 24 h. Finally, the viabilities of the SW620 cells were evaluated with an MTT assay. The untreated SW620 cells were used as a control. The relative cell viabilities (%) were calculated relative to the control value using the optical densities. The cell viabilities (%) of the Fe-CPND-stained cells, including the tumour cells (SW620 and HepG2) and normal tissue cells (HL-7702 and MC3T3-E1), were also measured with an MTT assay as previously described (except for the use of 808-nm NIR laser irradiation). For live/dead-cell staining using the calcein acetoxymethyl ester and propidium iodide stains, the cells were seeded in 96-well plates. After being incubated with Fe-CPNDs and irradiated by an 808-nm NIR laser as described above, the cells were stained with both calcein acetoxymethyl ester and propidium iodide and imaged by confocal fluorescence microscopy (Leica, CLSM TCS-SP2, Wetzlar, Germany).

For *in vitro* magnetic resonance imaging, the cells were incubated in 2 ml fresh L-15 containing various amounts of Fe-CPNDs and incubated at 37 °C for 12 h. The Fe-CPND-stained cells were washed with 1 ml PBS (three times), detached with 1 ml trypsin/EDTA from a cell-detaching kit (Gibco) and centrifuged at 2,000 r.p.m. for 5 min. The supernatants were then discarded, and the cell pellets were collected. The MRI was performed using a Siemens 1.5 T MRI scanner (Magnetom Avanto, Siemens) with the following imaging parameters: repetition time, 613 ms; echo time, 8.4 ms; field of view, 102 × 72 mm; and slice thickness, 3.0 mm.

### *In vivo* MRI and photothermal therapy

Male BALB/nu mice (6 weeks old, average weight 20 g) were purchased from Beijing Vital River Laboratory Animal Technology Co., Ltd, (Beijing, China). Mice were maintained in a controlled environment with a 12-h/12-h light/dark cycle and provided with food and water *ad libitum*. The animal procedures followed the guidelines of the regional ethics committee for animal experiments established by Jilin University Institutional Animal Care and Use. The tumour models were established by subcutaneously inoculating nude mice with 5 × 10^6^ SW620 cells suspended in 100 μl of serum-free L-15.

To investigate the MRI contrast of Fe-CPNDs in the tumour, two nude mice selected at random were anaesthetized using 100 μl chloral hydrate (10 wt%). One hundred microlitres of NaCl solution (0.9 wt%) containing the desired amount (0.25 mg kg^−1^) of Fe-CPNDs was injected into the tumour. MRI was performed at the desired time points after injection. The *T*_1_-weighted magnetic resonance images were acquired using a Siemens 1.5 T MRI scanner with the following imaging parameters: repetition time, 358 ms; echo time, 15 ms; field of view, 120 × 72 mm; and slice thickness, 2.0 mm. To determine the ability of the pH to activate the NPs *in vivo*, Fe-CPNDs (0.35 mg kg^−1^) were injected subcutaneously into the tumour site and thigh muscles. For the *in vivo* tumour accumulation study, 80 μl Fe-CPNDs of various concentrations (2, 0.8 and 0.2 mg kg^−1^) were injected intravenously into randomly selected mice (*n*=2 per group). For the biodistribution analysis, 80 μl Fe-CPNDs (0.8 mg kg^−1^) was intravenously injected into a normal mouse selected at random (*n*=2). MRI was then performed as previously described.

For the photothermal therapy, when the tumour size reached 5 mm in diameter, the nude mice were divided into four randomized groups and treated with PBS only (group I), Fe-CPNDs only (group II), PBS and 808-nm NIR laser irradiation for 6 min (group III) and Fe-CPNDs and 808-nm NIR laser irradiation for 6 min (group VI). For group VI, the nude mice were intravenously injected with 100 μl Fe-CPND aqueous dispersions of various concentrations (0.5, 0.2, and 0.1 mg kg^−1^). Each group consisted of four mice. The tumours were irradiated with an 808-nm laser at 1.3 W cm^−2^ for 6 min 2.5 h after injection. The control group was not irradiated. The tumour dimensions were measured with a calliper for 20 days. The tumour volume was calculated according to the following formula: tumour volume=(tumour length) × (tumour width)^2^/2. The relative tumour volumes were calculated as follows: *V*/*V*_0_ (*V*_0_ is the tumour volume when the treatment was initiated).

### Toxicology analysis

For the *in vivo* toxicity studies, a NaCl solution (0.9 wt%) containing Fe-CPNDs containing 5 mg kg^−1^ Fe content was administered intravenously to healthy mice. Over 1 month, the mice were observed for behavioural changes and weighed daily. The mice were then killed, and the tissues (including the heart, spleen, liver, lung, kidney, cerebrum, cerebellum and testis) were fixed in 10% neutral buffered formalin 30 days after injection. The histological sections were observed under an optical microscope. For the blood analysis, the healthy group was intravenously administered a single dose of the nanocomposite, and the untreated group was used as a control. One month later, the blood was extracted for the haematology analysis and blood biochemistry assay.

### Statistical analysis

Quantitative data are expressed as the mean±s.d. For the MRI, each nude mouse was scanned five times to calculate the mean value and s.d.. In the biodistribution study, each sample was divided into five equal parts and measured by ICP-MS. All statistical analyses were performed using the Excel software (Analysis ToolPak for Microsoft Excel 2010). Comparisons between the two groups were made based on an analysis of variance and Tukey's *post hoc* test using the ORIGIN software. *P* value of <0.05 and 0.01 was considered to indicate significant differences.

## Additional information

**How to cite this article:** Liu, F. *et al*. Gram-scale synthesis of coordination polymer nanodots with renal clearance properties for cancer theranostic applications. *Nat. Commun.* 6:8003 doi: 10.1038/ncomms9003 (2015).

## Supplementary Material

Supplementary Information Supplementary Figures 1-43, Supplementary Tables 1-3, Supplementary Discussion, Supplementary Methods and Supplementary References
