# Supplementary material for: Gram-scale synthesis of coordination polymer nanodots with renal clearance properties for cancer theranostic applications
Source: Nat Commun. 2015 Aug 6;6:8003. doi: 10.1038/ncomms9003 (PMC4918390; doi:10.1038/ncomms9003)
Supplement: Supplementary Information — Supplementary Figures 1-43, Supplementary Tables 1-3, Supplementary Discussion, Supplementary Methods and Supplementary References [file ncomms9003-s1.pdf]

Supplementary Figures 1-43 and Table 1-3

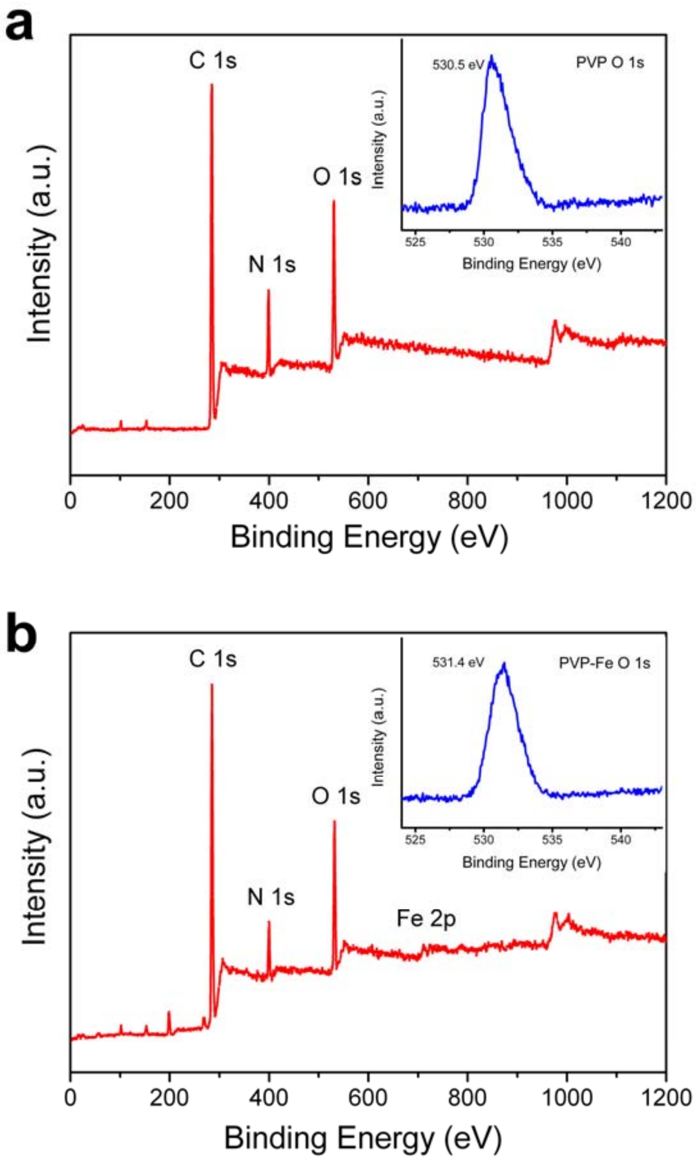

Supplementary Figure 1 | XPS characterization. XPS survey spectra of (a) PVP and (b) PVP-Fe. The insets show the corresponding O 1s XPS spectra.

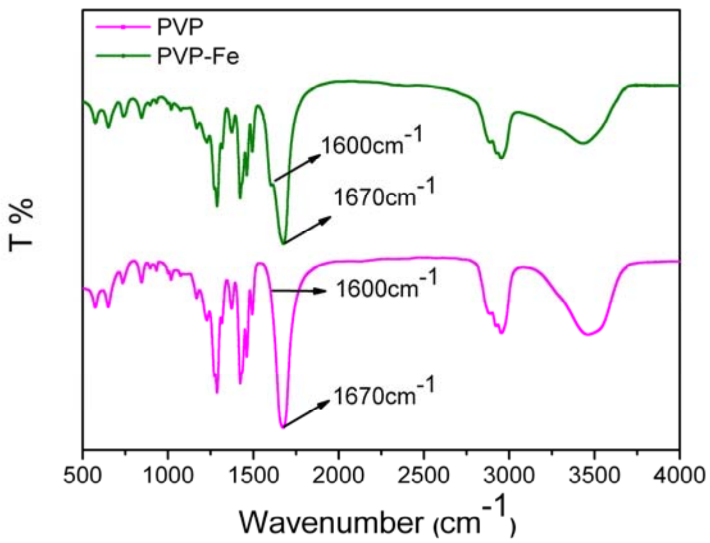

Supplementary Figure 2 | FTIR characterization. FTIR spectra of PVP and PVP-Fe, respectively.

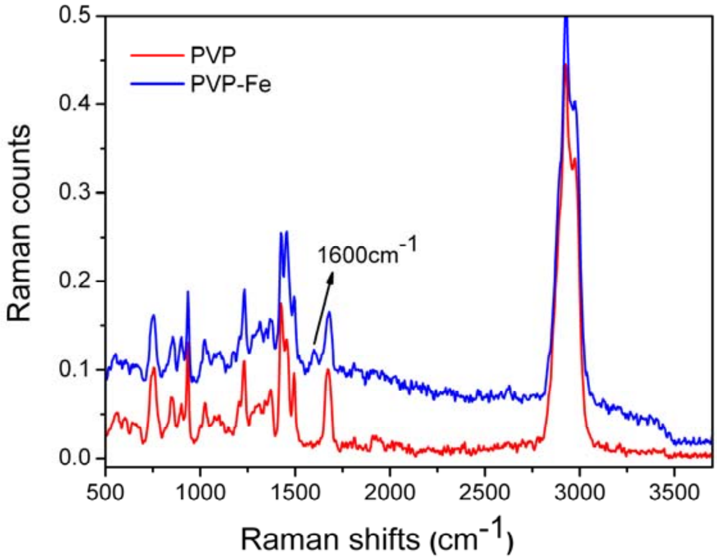

Supplementary Figure 3 | Fourier transform Raman characterization. Fourier transform Raman spectra of PVP and PVP-Fe, respectively. The Raman spectra were recorded with a FT-Raman spectrometer (Thermo Nicolet 960) equipped with an InGaAs detector and a Nd/VO4 laser (1064 nm) as an excitation source.

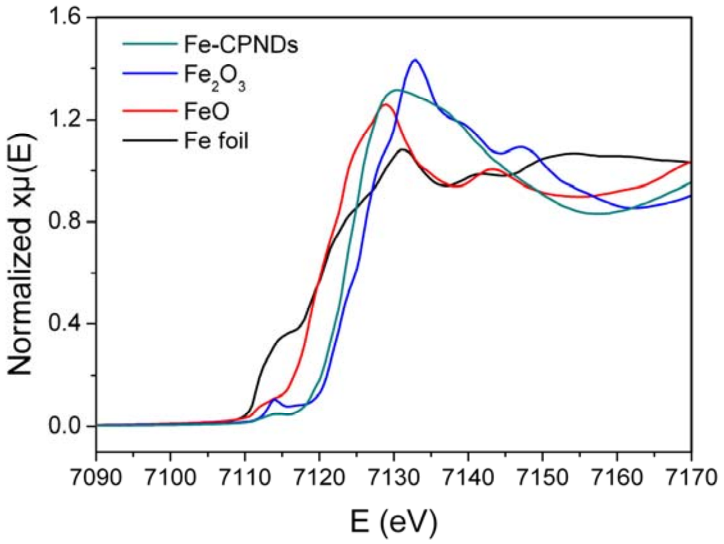

Supplementary Figure 4 | XANES characterization. Fe K-edge XANES spectra of the Fe-CPNDs and models.

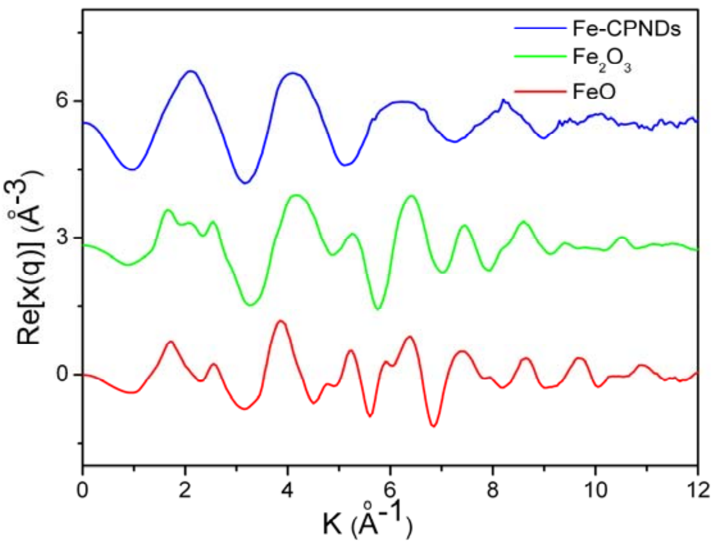

Supplementary Figure 5 | k^2-weighted EXAFS spectra. Fourier transform magnitude of k^2-weighted Fe K-edge EXAFS spectra of the Fe-CPNDs and references.

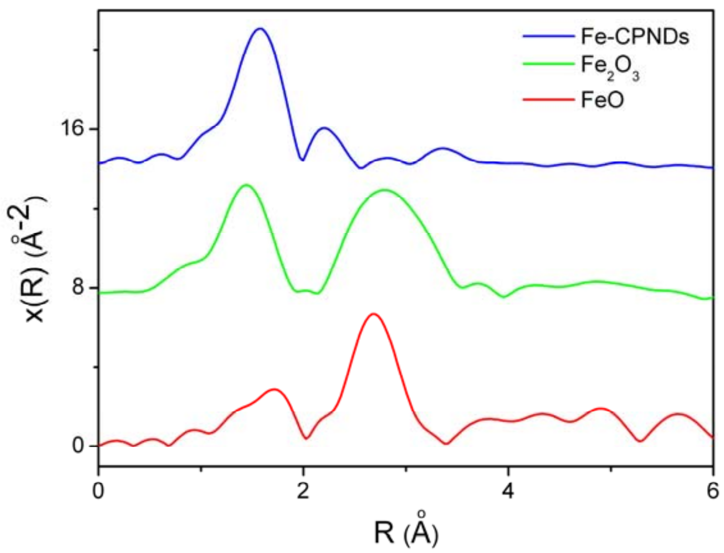

Supplementary Figure 6 | k^3-weighted EXAFS spectra. Fourier transform magnitude of k^3-weighted Fe K-edge EXAFS spectra of the Fe-CPNDs and references.

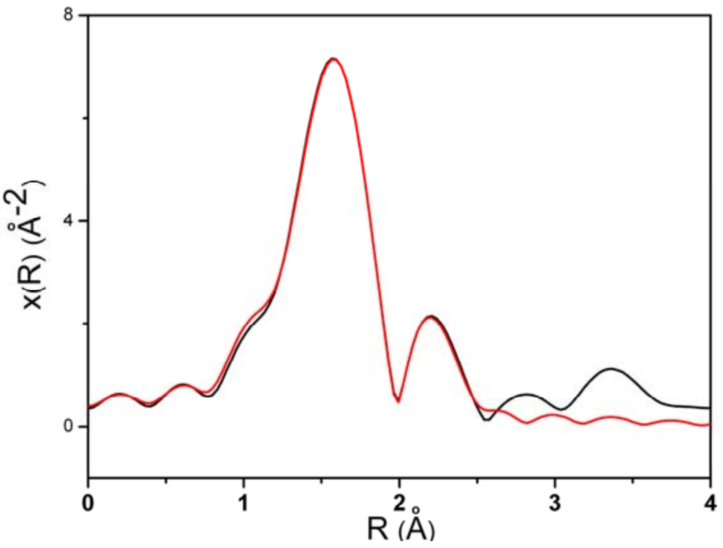

Supplementary Figure 7 | k^3-weighted EXAFS spectra of Fe-CPNDs. Fourier transform magnitude of k^3-weighted Fe K-edge EXAFS spectra of the Fe-CPNDs. Experimental (black line) and Fit (red line).

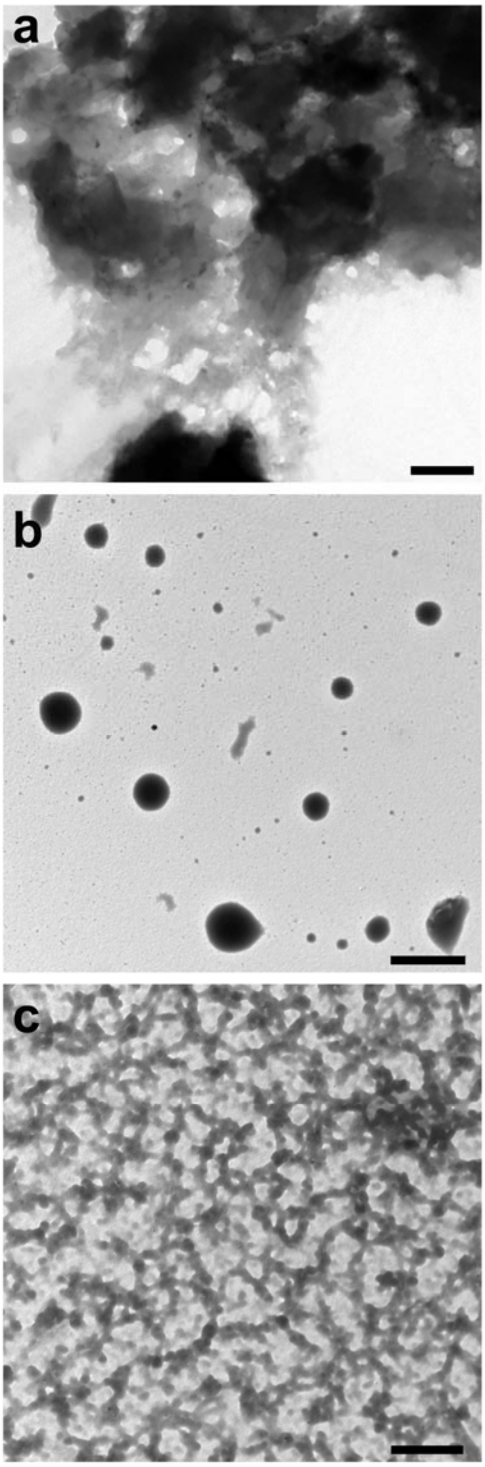

Supplementary Figure 8 | TEM characterization. TEM micrographs of (a) GA-Fe; (b) PVP-GA and (c) PVP-Fe, respectively. Scale bar, 200 nm.

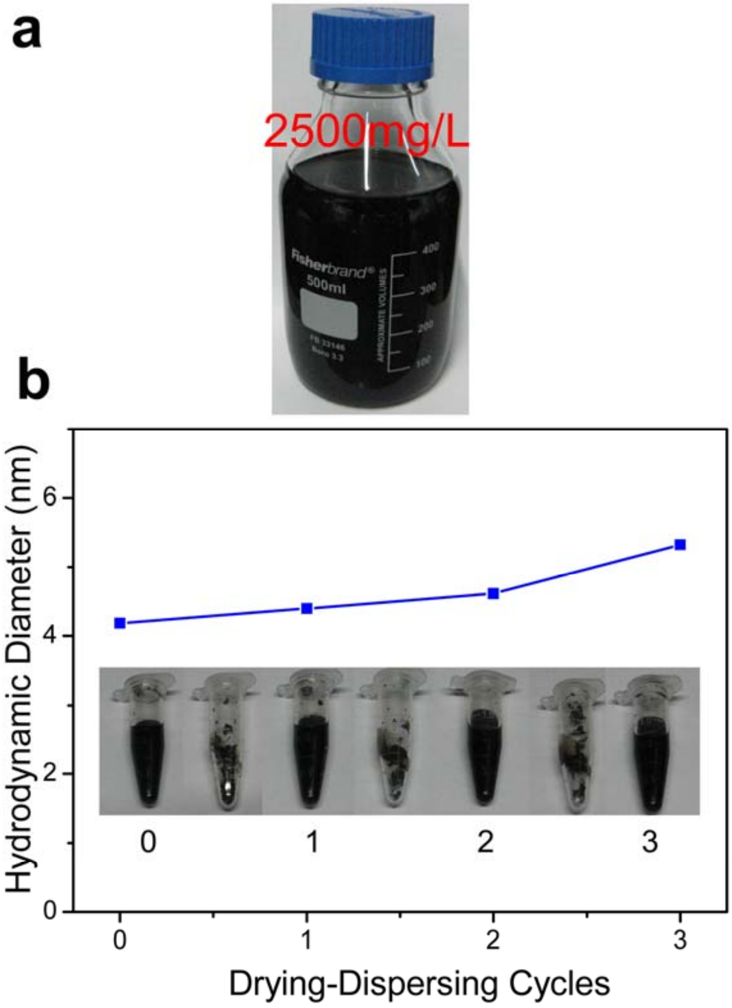

Supplementary Figure 9 | The digital photograph and DLS characterization of Fe-CPNDs. (a) The digital photograph of one-pot synthesized Fe-CPNDs. (b) DLS measurements of Fe-CPNDs after multiple dried-dissolved processes. The inset shows the digital photographs of Fe-CPNDs after multiple dried-dissolved processes.

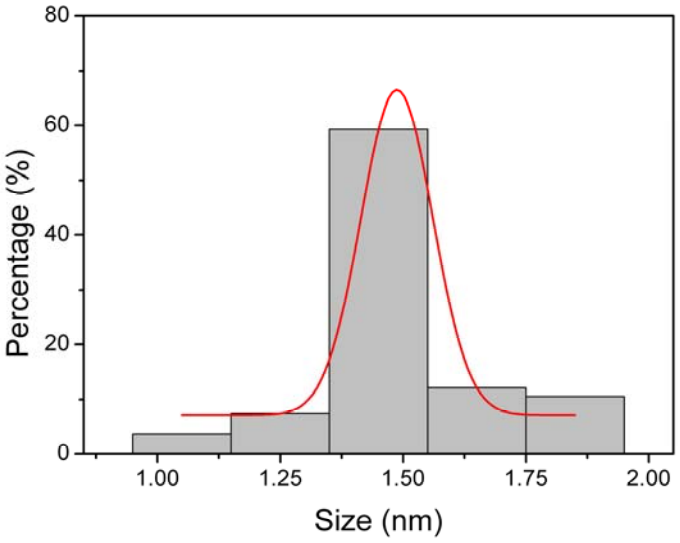

Supplementary Figure 10 | The size distribution characterization of Fe-CPNDs. The size distribution of Fe-CPNDs from TEM observations. 100 Fe-CPNDs in the TEM micrographs were arbitrarily selected for the size distribution.

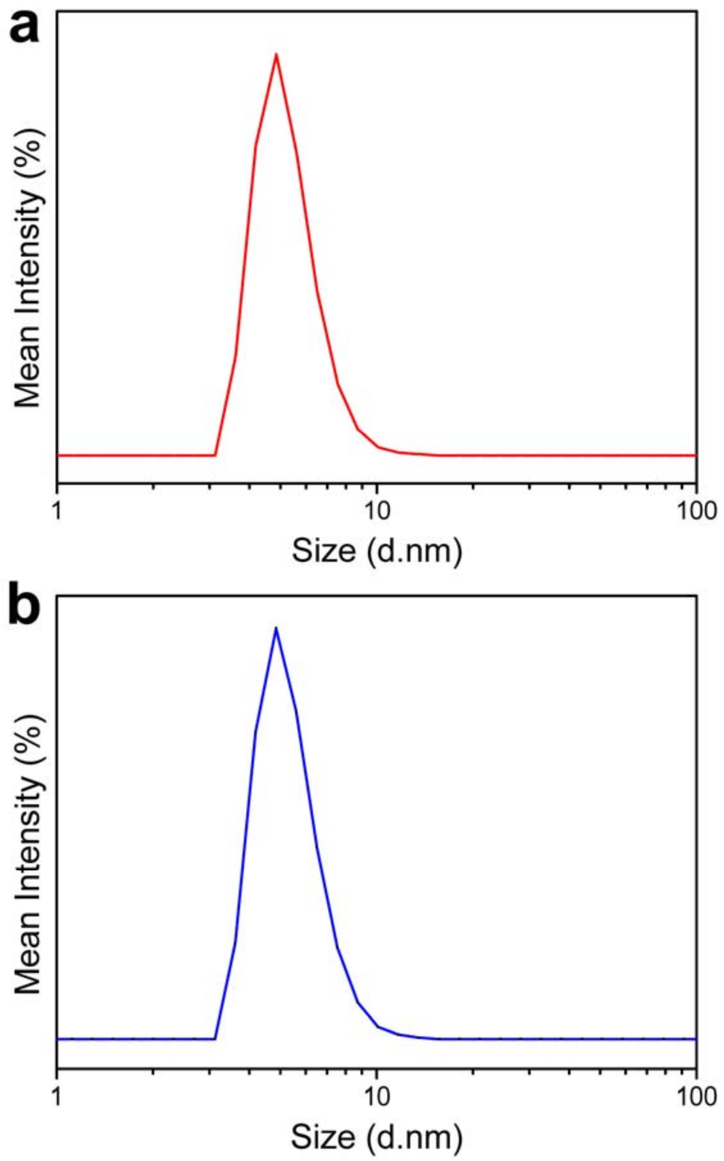

Supplementary Figure 11 | DLS characterization of Fe-CPNDs. DLS measurements of Fe-CPNDs in (a) PBS buffer and (b) fetal bovine serum solution (10%, v/v).

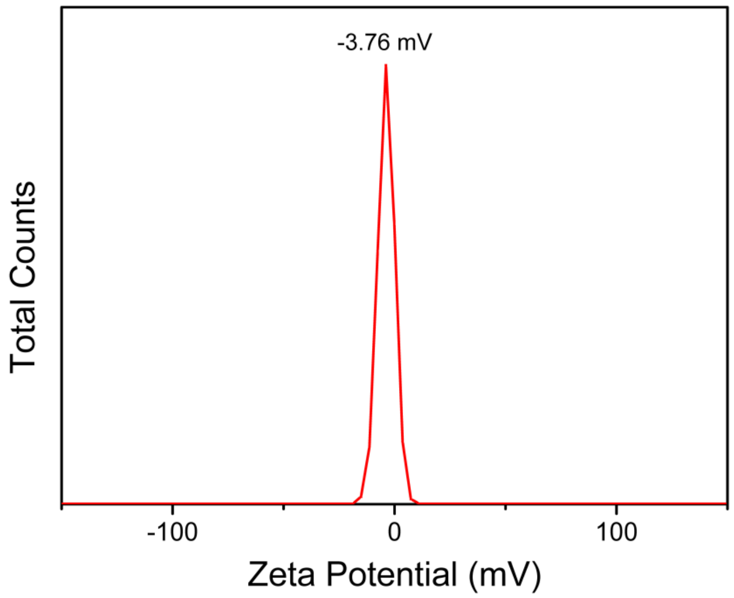

Supplementary Figure 12 | Zeta Potential characterization of Fe-CPNDs. Zeta Potential measurement of Fe-CPNDs.

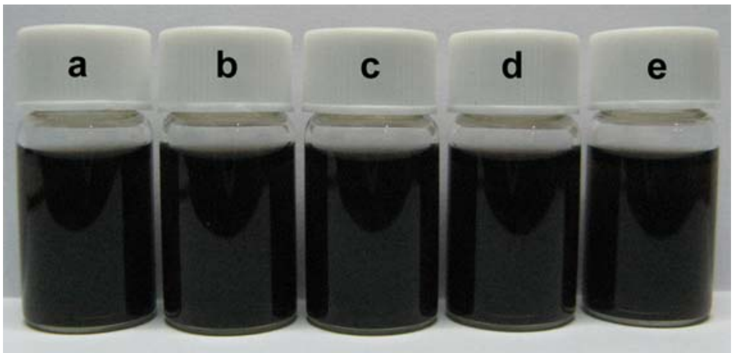

Supplementary Figure 13 | The stability characterization of Fe-CPNDs. Dispersibility of Fe-CPNDs in different media including (a) H_2O, (b) PBS (pH=7.4, 100 mM), (c) TB (pH=8.5, 100 mM), (d) 0.9% NaCl solution and (e) DMEM supplemented with 10% FBS (v/v), respectively.

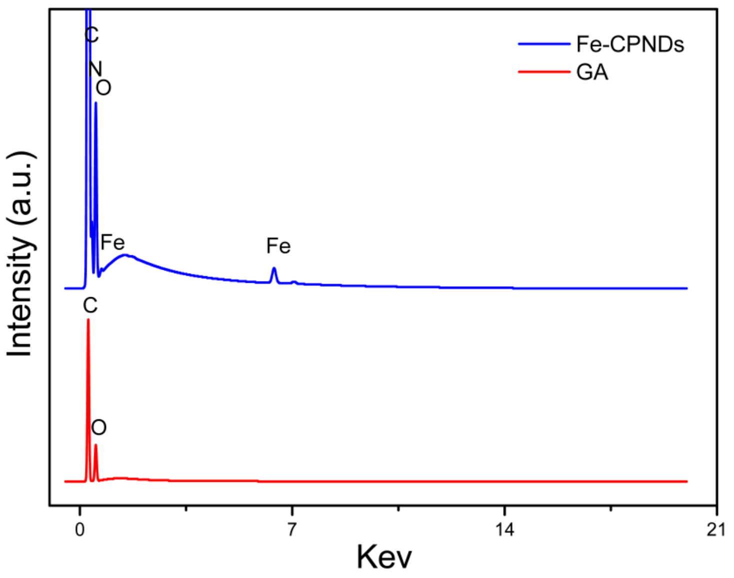

Supplementary Figure 14 | EDS characterization. EDS analysis of Fe-CPNDs and GA, respectively.

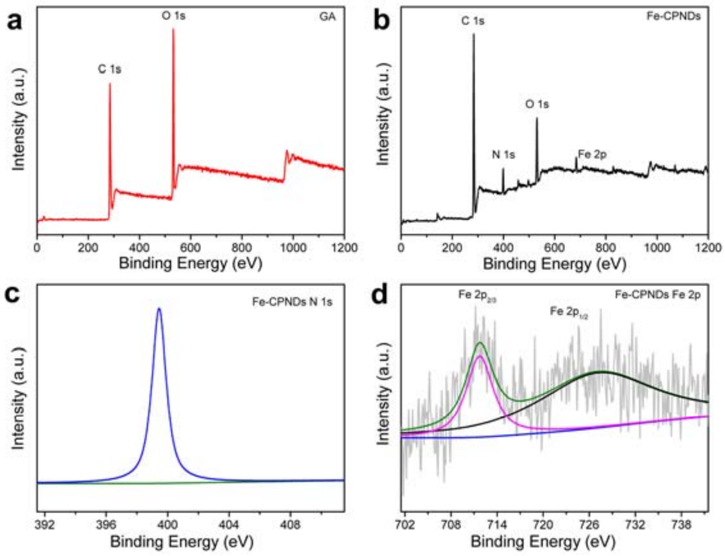

Supplementary Figure 15 | XPS characterization. (a) and (b) XPS survey spectra of GA and Fe-CPNDs, respectively. (c) and (d) N 1s and Fe 2p XPS spectra of Fe-CPNDs, respectively.

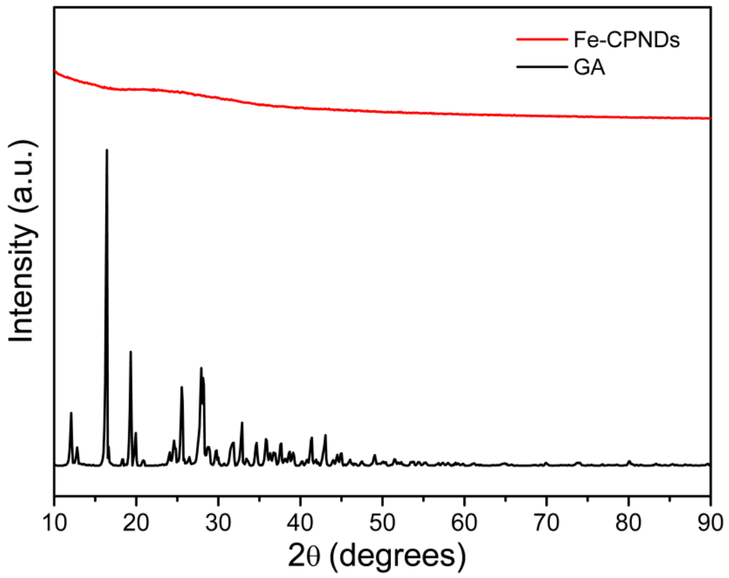

Supplementary Figure 16 | Powder XRD characterization. Powder XRD patterns of Fe-CPNDs and GA, respectively.

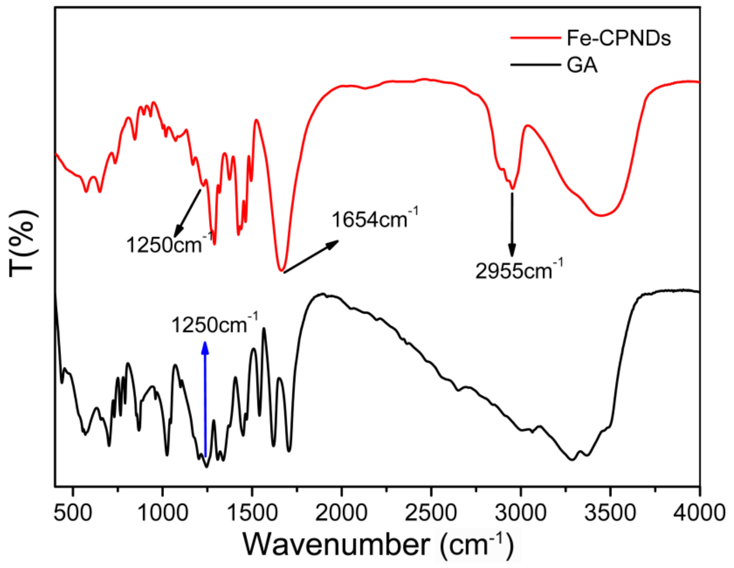

Supplementary Figure 17 | FTIR characterization. FTIR spectra of Fe-CPNDs and GA, respectively.

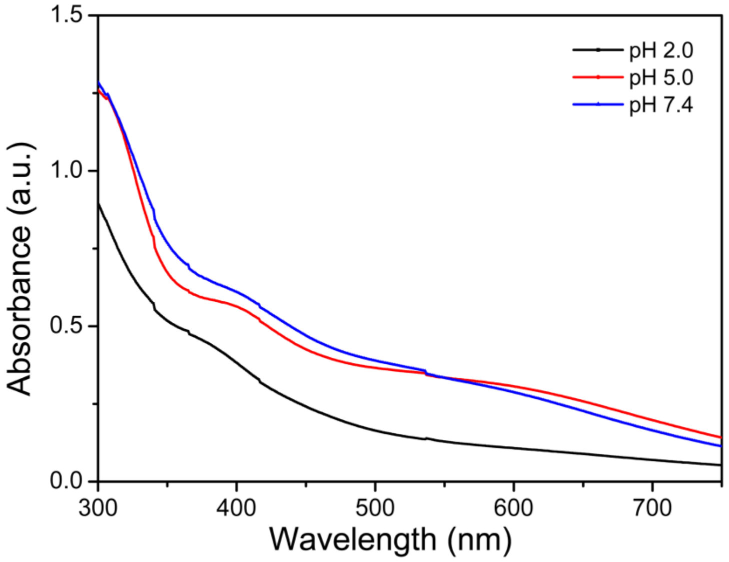

Supplementary Figure 18 | UV-visible spectra of Fe-CPNDs. UV-visible spectra of Fe-CPNDs solutions under different pH values.

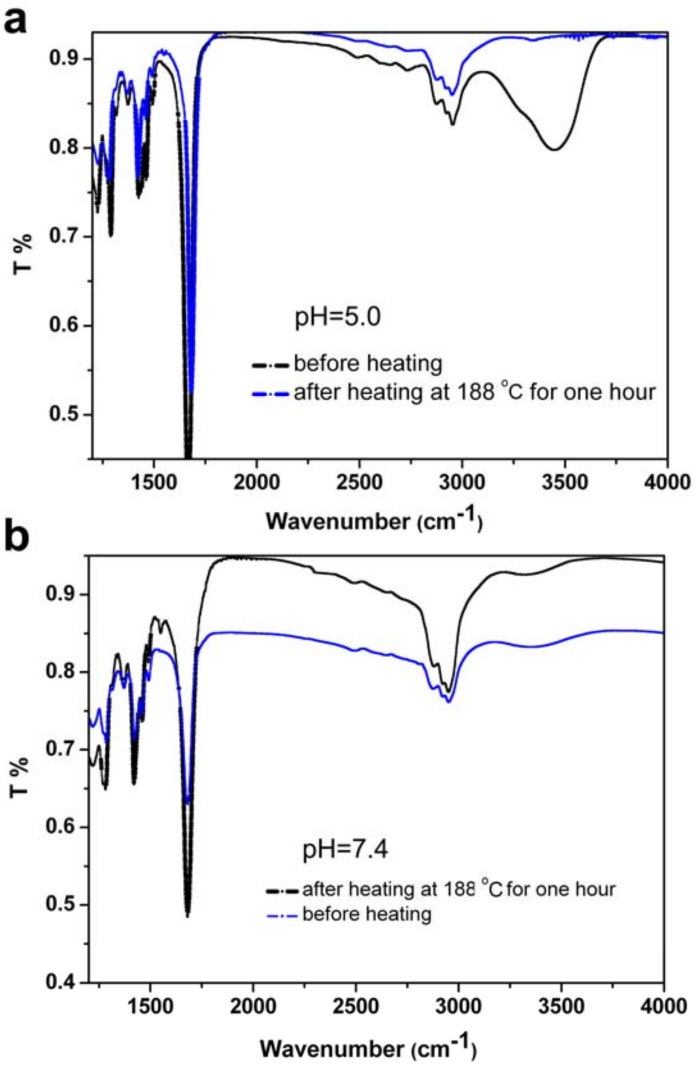

Supplementary Figure 19 | FTIR characterization at different temperatures. FTIR spectra of Fe-CPNDs at different temperatures. The Fe-CPNDs were incubated at 37 °C for two hours at different pH buffers and dried in vacuum at 60 °C for 12 h before FTIR measurement.

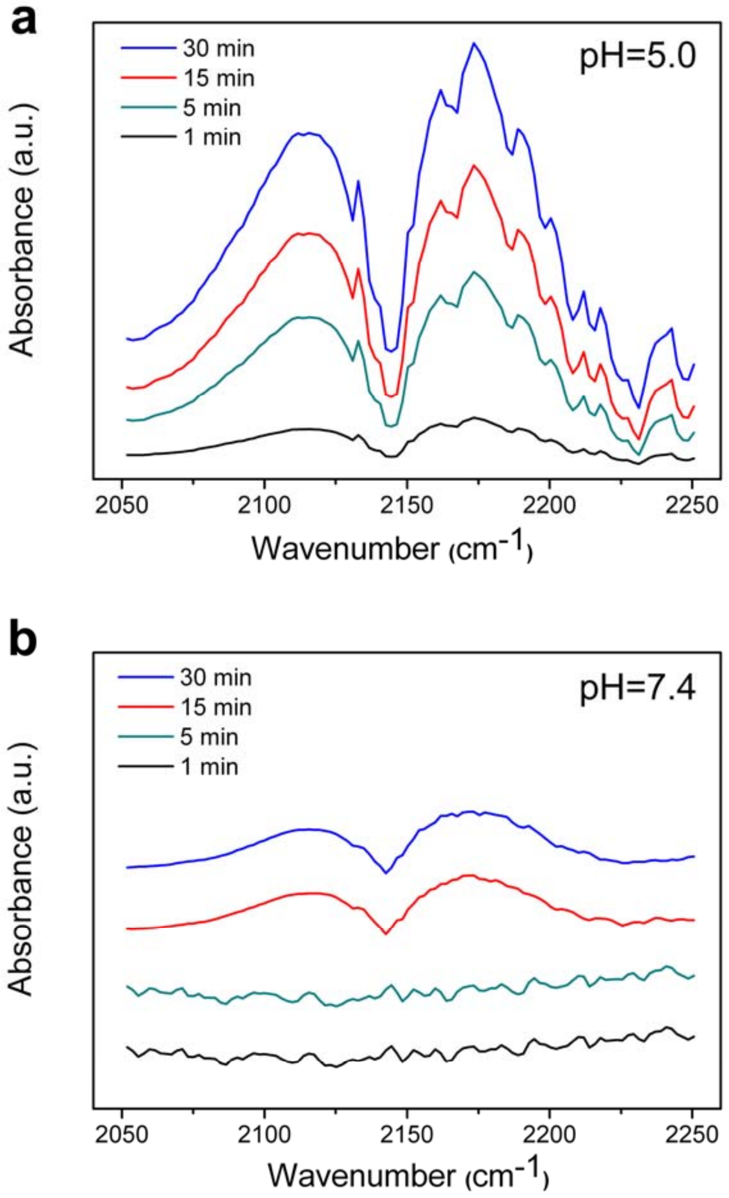

Supplementary Figure 20 | FTIR characterization of CO adsorbed on Fe-CPNDs.

FTIR spectra of CO adsorbed on Fe-CPNDs. The Fe-CPNDs were incubated at 37 ^{o}C for two hours at different pH buffers before FTIR measurement.

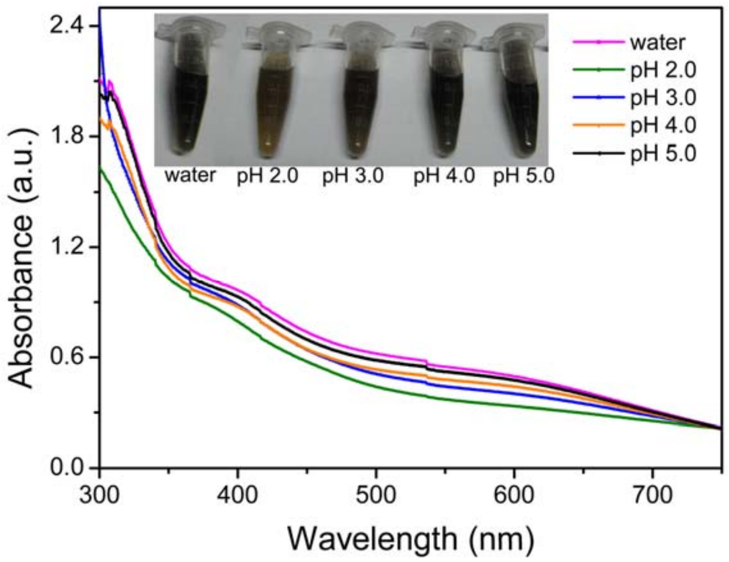

Supplementary Figure 21 | UV-visible spectra of Fe-CPNDs. UV-visible spectra of Fe-CPNDs solutions under different pH values and incubated at 37 ^{o}C for 24 h. The inset shows the corresponding digital photographs of Fe-CPNDs solutions after the incubation.

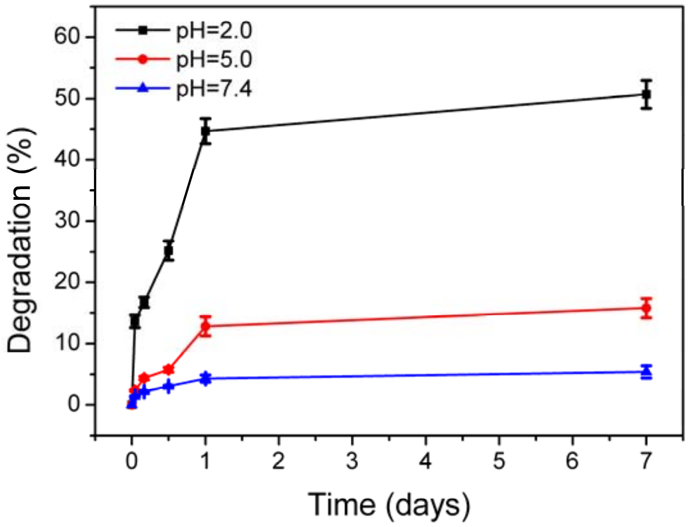

Supplementary Figure 22 | The degradation characterization of Fe-CPNDs.

Release of GA from Fe-CPNDs which were incubated at various pH values.

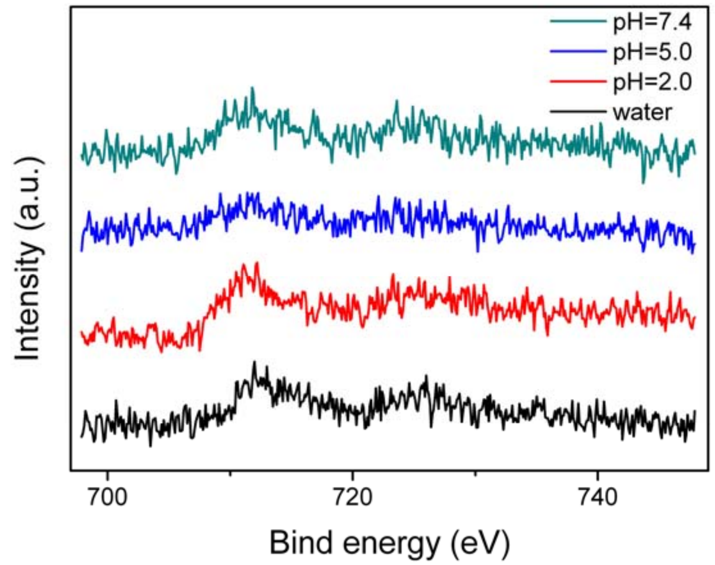

Supplementary Figure 23 | XPS characterization of Fe-CPNDs. Fe 2p XPS spectra of Fe-CPNDs after incubation at various pH values for 1 week.

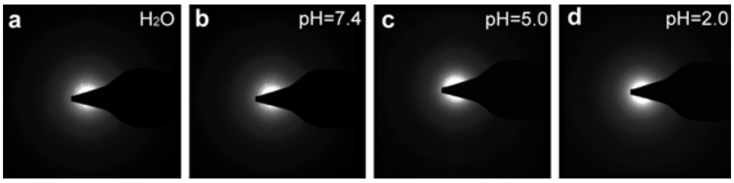

Supplementary Figure 24 | FFT patterns of Fe-CPNDs. FFT patterns of Fe-CPNDs after incubation at various pH values for 1 week.

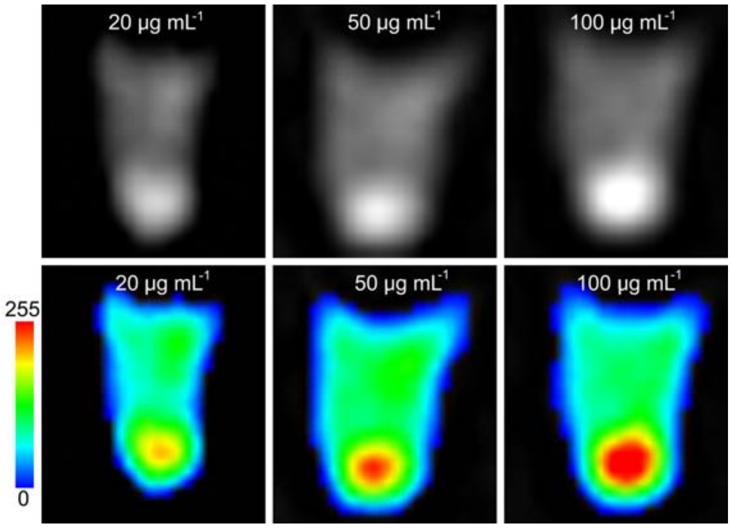

Supplementary Figure 25 | *In vitro* MRI images of Fe-CPNDs. MRI images of Fe-CPNDs stained SW620 cells at various concentrations (Fe content).

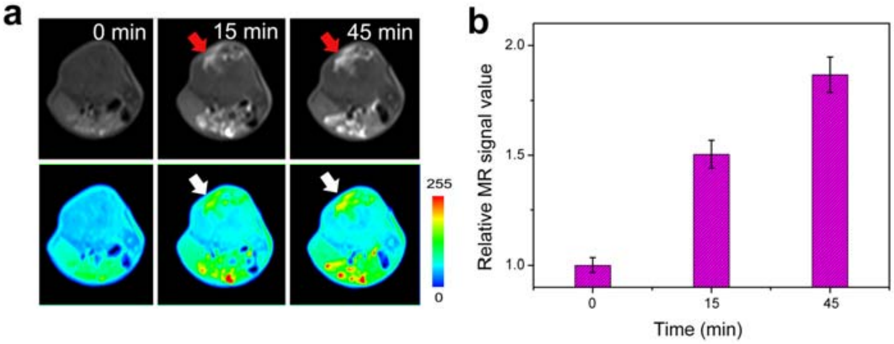

Supplementary Figure 26 | *In vivo* MRI imaging of colorectal tumor xenografts after intratumor injection. (a) *In vivo* MR images of selected nude mouse bearing colorectal tumor (SW620) after intratumor injection of Fe-CPNDs at different time intervals (The time 0 min means pre-injection.). The injection sites of tumor were showed by red and white arrows. (b) Corresponding data analysis of tumor in (a). Error bars mean standard deviations (n=5). Each nude mouse was repeated scanning 5 times for calculated standard deviations. The parallel model of Figure 3a.

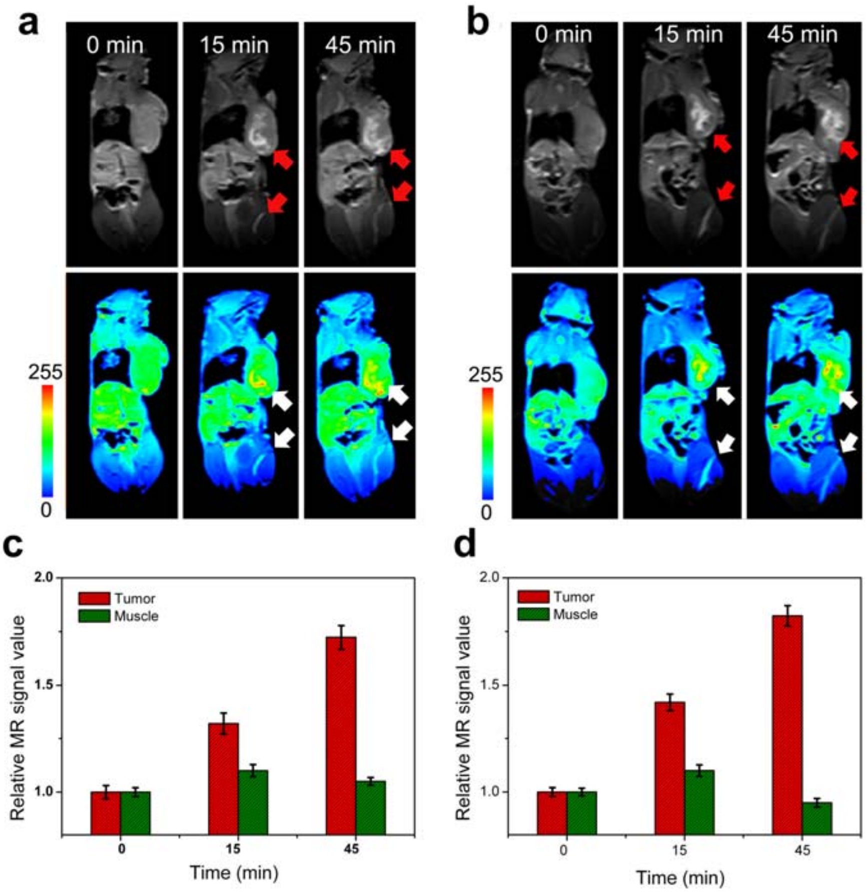

Supplementary Figure 27 | *In vivo* MRI imaging of colorectal tumor xenografts in tumor and normal subcutaneous tissue after injection. (a, b) *In vivo* MR images of selected 2 nude mice bearing colorectal tumor (SW620) after injection of Fe-CPNDs within tumor and normal subcutaneous tissue (thigh muscle) at different time intervals (The time 0 min means pre-injection.). The injection sites of tumor and muscle were showed by red and white arrows, respectively. (c) Corresponding data analysis of tumor and thigh muscle in (a). (d) Corresponding data analysis of tumor and thigh muscle in (b). Error bars mean standard deviations (n=5), each nude mouse was repeated scanning 5 times for calculated standard deviations. The comparison models of pH-activatable experiment *in vivo*.

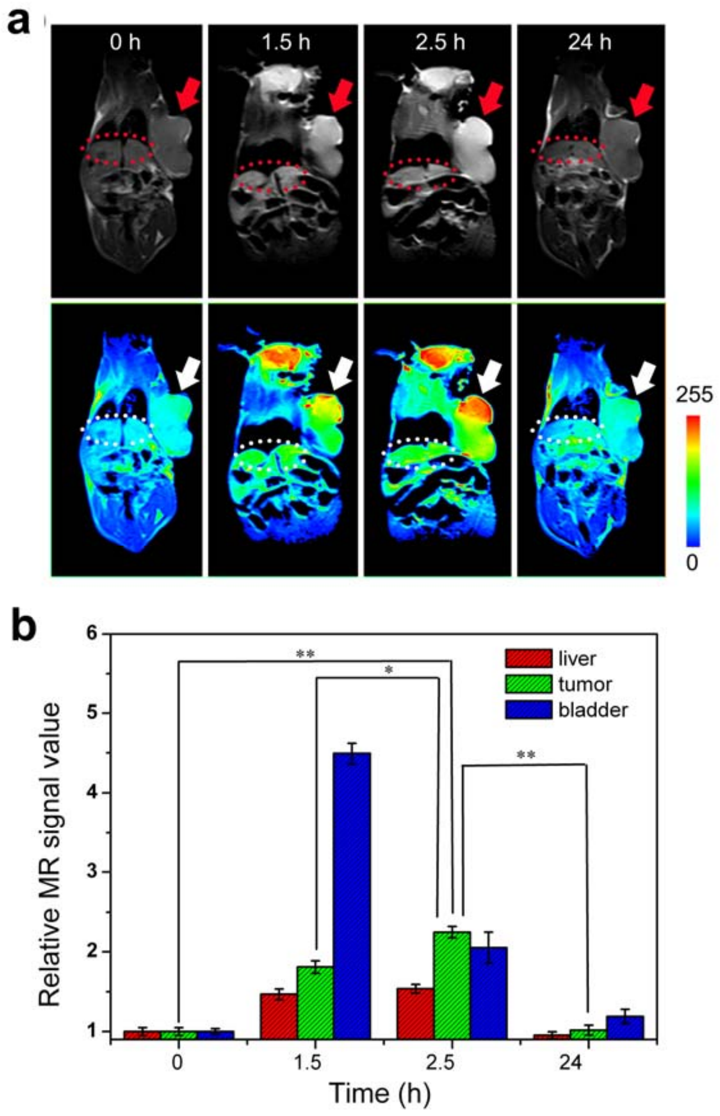

Supplementary Figure 28 | *In vivo* MRI imaging of colorectal tumor xenografts. *In vivo* MR images and data analysis of selected nude mouse bearing colorectal tumor after intravenous injection (tumor, arrows; liver, ellipses) of Fe-CPNDs at different time intervals (The time 0 h means pre-injection.). Error bars mean standard deviations (n=5, $^{**}p < 0.01$, or $^{*}p < 0.05$, by ANOVA with Tukey's post-test). Each nude mouse was repeated scanning 5 times for calculated standard deviations. The parallel model of Figure 3b.

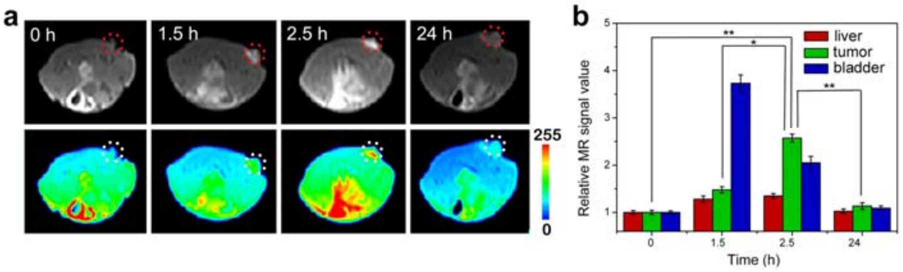

Supplementary Figure 29 | *In vivo* MRI imaging of tiny colorectal tumor xenografts. (a) *In vivo* MR images nude mice bearing tiny (*ca.* 5 mm^3 in volume), colorectal tumor after intravenous injection (tumor, circles) of Fe-CPNDs at different time intervals (The time 0 h means pre-injection.). (b) Corresponding data analysis of tumor and main organs in (a). Error bars mean standard deviations (n=5, **p < 0.01, or *p < 0.05, by ANOVA with Tukey's post-test). Each nude mouse was repeated scanning 5 times for calculated standard deviations. The parallel model of Figure 4.

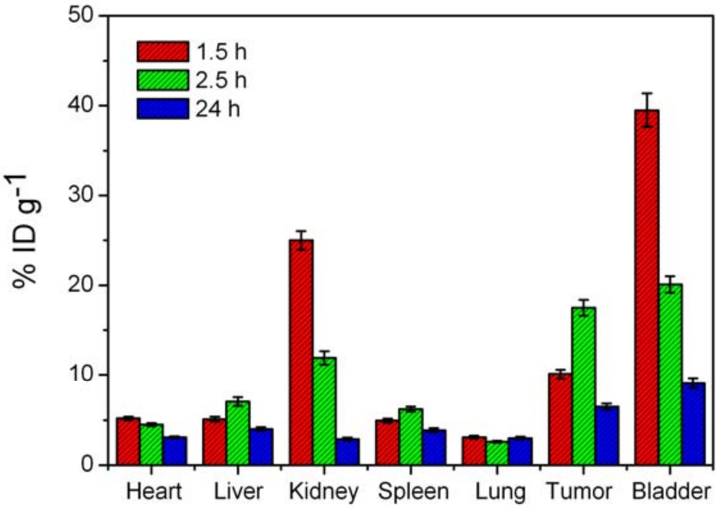

Supplementary Figure 30 | *In vivo* Biodistribution analysis of Fe-CPNDs after intravenous injection. Biodistribution of Fe-CPNDs in tumor and main organs of mice after intravenous injection at different time intervals. Error bars mean standard deviations (n=5).

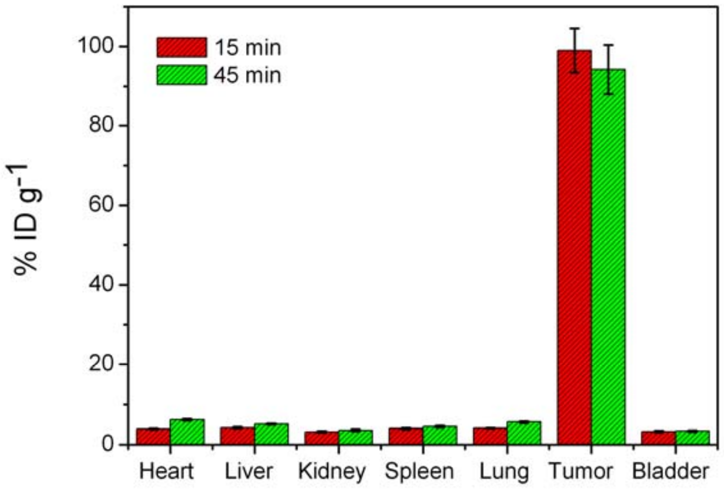

Supplementary Figure 31 | *In vivo* Biodistribution analysis of Fe-CPNDs after intratumor injection. Biodistribution of Fe-CPNDs in tumor and main organs of mice after intratumor injection at different time intervals. Error bars mean standard deviations (n=5).

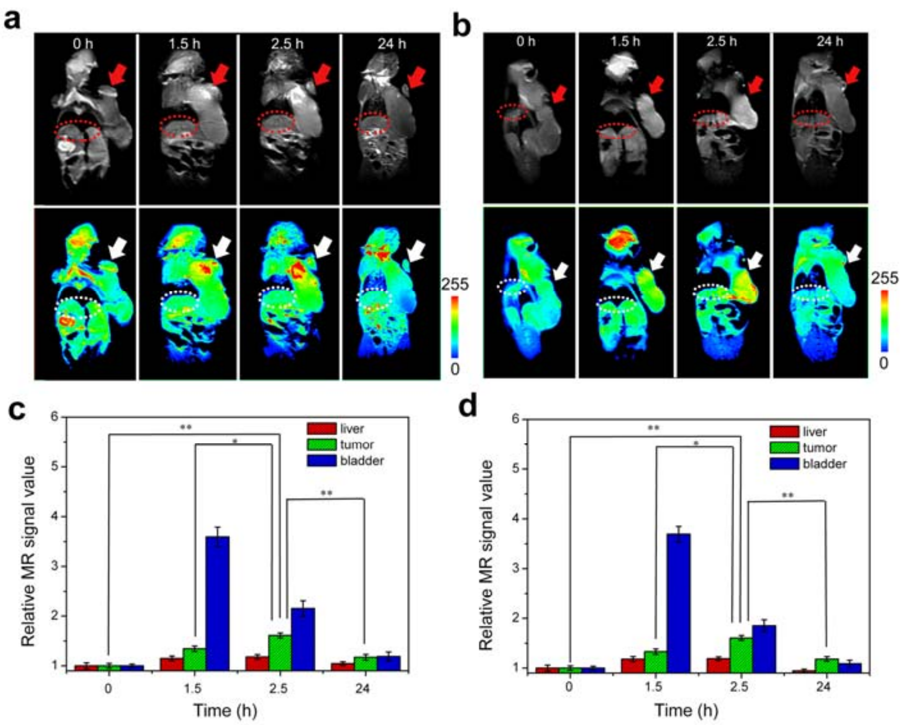

Supplementary Figure 32 | *In vivo* MRI imaging of colorectal tumor xenografts after intravenous injection at lower dose. (a, b) *In vivo* MR images of selected 2 nude mice bearing colorectal tumor (SW620) after intravenous injection (tumor, arrows; liver, ellipses) of Fe-CPNDs (0.8 mg kg^{-1}, 100 μL) at different time intervals (The time 0 h means pre-injection.). (c) Corresponding data analysis of tumor and main organs in (a). (d) Corresponding data analysis of tumor and main organs in (b). Error bars mean standard deviations.(n=5, **p < 0.01, or *p < 0.05, by ANOVA with Tukey's post-test). Each nude mouse was repeated scanning 5 times for calculated standard deviations.

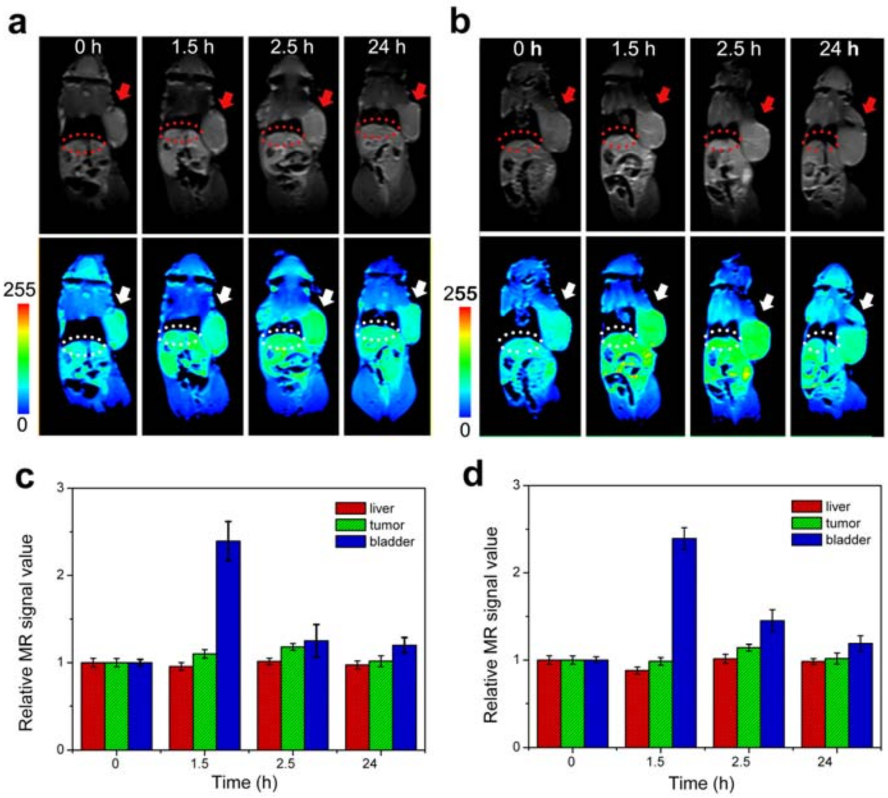

Supplementary Figure 33 | *In vivo* MRI imaging of colorectal tumor xenografts after intravenous injection at lowest dose. (a, b) *In vivo* MR images of selected 2 nude mice bearing colorectal tumor (SW620) after intravenous injection (tumor, arrows; liver, ellipses) of Fe-CPNDs (0.2 mg kg^{-1}, 80 μL) at different time intervals (The time 0 h means pre-injection.). (c) Corresponding data analysis of tumor in (a). (d) Corresponding data analysis of tumor in (b). Error bars mean standard deviations.(n=5, **$p < 0.01$, or *$p < 0.05$, by ANOVA with Tukey's post-test). Each nude mouse was repeated scanning 5 times for calculated standard deviations.

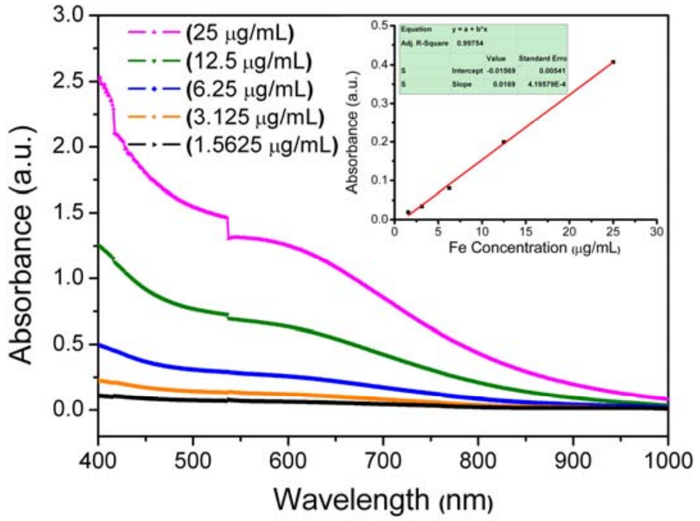

Supplementary Figure 34 | UV-visible spectra of Fe-CPNDs at different concentrations. UV-visible spectra of Fe-CPNDs at different concentrations (Fe content: 0-25 μg mL^{-1}). The inset shows a linear relationship for the optical absorbance at 808 nm as a function of the concentration of Fe-CPNDs.

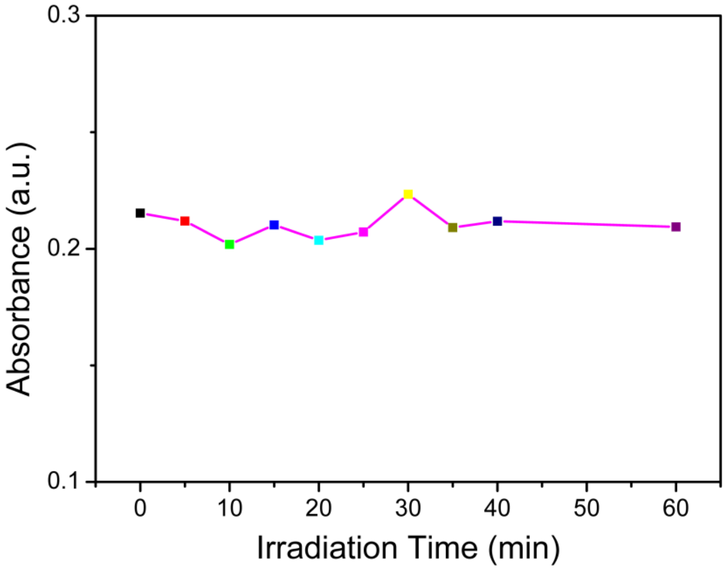

Supplementary Figure 35 | Photothermal stability analysis of Fe-CPNDs. The absorbance of Fe-CPNDs at 808 nm as a function of 808 nm NIR laser irradiation time.

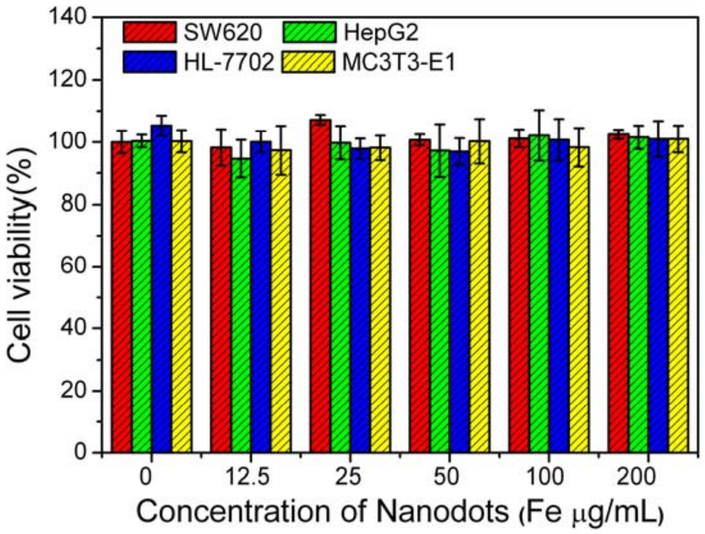

Supplementary Figure 36 | MTT analysis of Fe-CPNDs. Cell viabilities of different cells incubated with various concentrations of Fe-CPNDs.

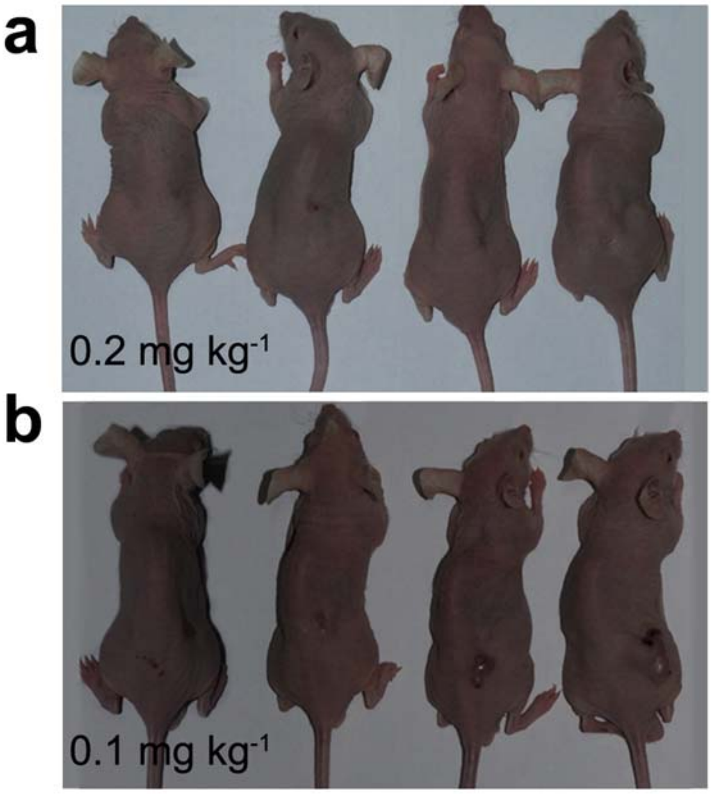

Supplementary Figure 37 | *In vivo* Photothermal therapy of Fe-CPNDs at different injection dose. Digital photographs of the mice at the end of intravenous treatments (day 20). The administered doses of Fe-CPNDs are (a) 0.2 mg kg^{-1} and (b) 0.1 mg kg^{-1}, respectively.

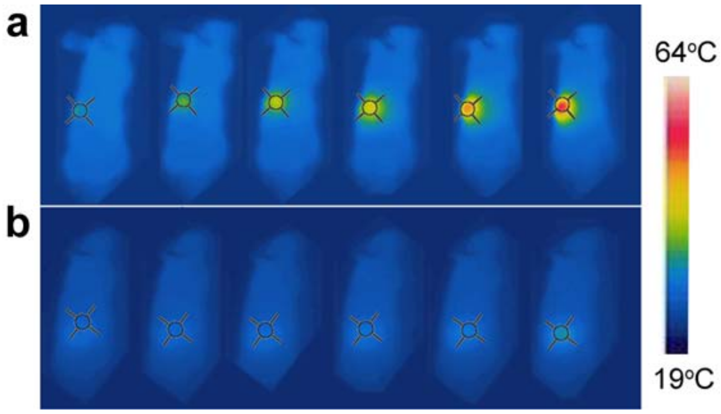

Supplementary Figure 38 | *In vivo* infrared thermal images analysis. *In vivo* infrared thermal images of SW620 tumor-bearing mouse after injection of (a) 0.2 mg kg^{-1} Fe-CPNDs and (b) 0.9% NaCl solution only as a function of the 808 nm NIR laser irradiation time (1.3 W cm^{-2}).

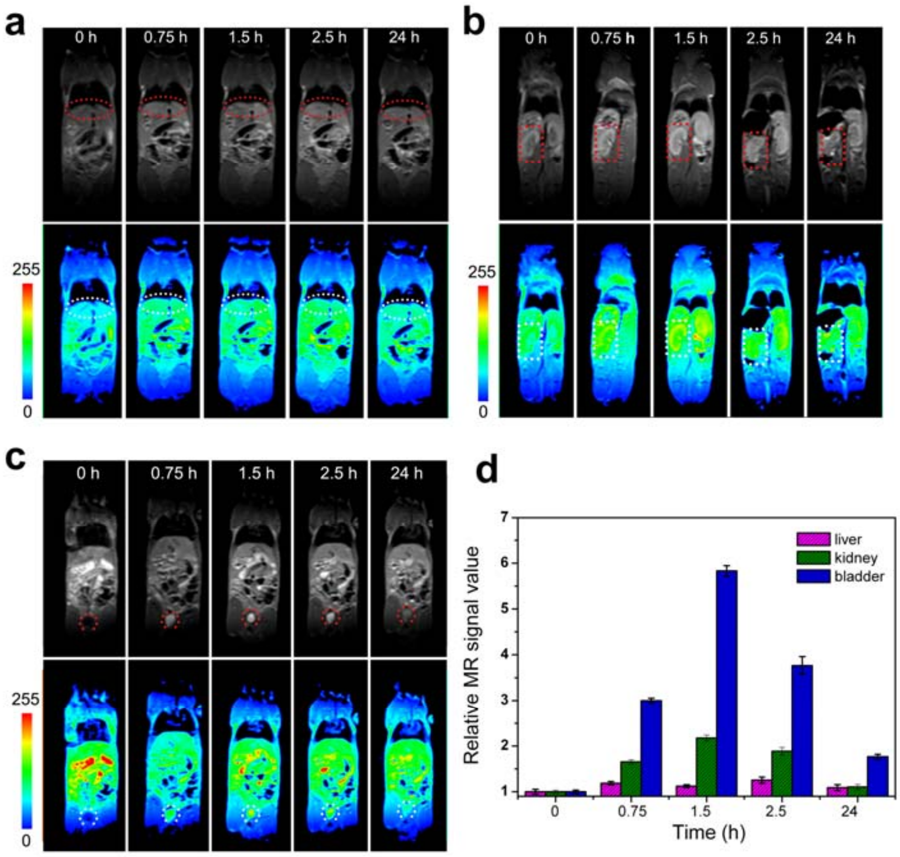

Supplementary Figure 39 | *In vivo* MRI imaging of different organs. *In vivo* MR images of (a) liver (ellipses), (b) kidney (rectangles) and (c) bladder (circles) after intravenous injection of Fe-CPNDs at different time intervals (The time 0 h means pre-injection). (d) Corresponding data analysis of the MR measurements. Error bars mean standard deviations. Each nude mouse was repeated scanning 5 times for calculated standard deviations. The parallel model of Figure 5.

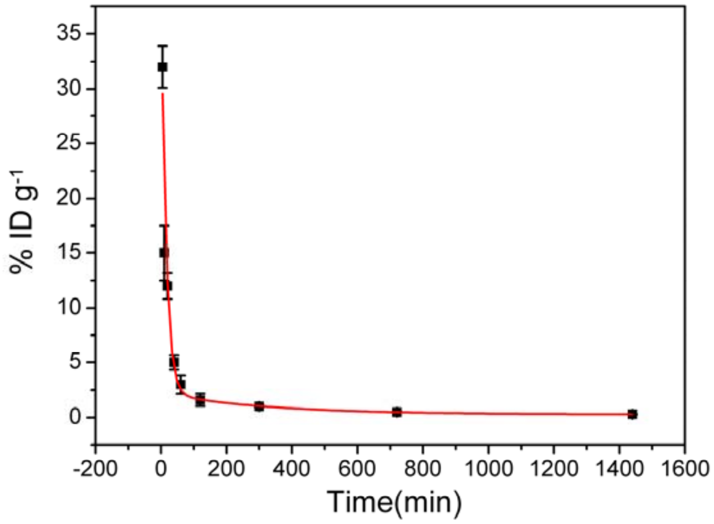

Supplementary Figure 40 | The pharmacokinetics analysis of Fe-CPNDs. The pharmacokinetics of Fe-CPNDs after intravenous injection. Error bars mean standard deviations (n=5).

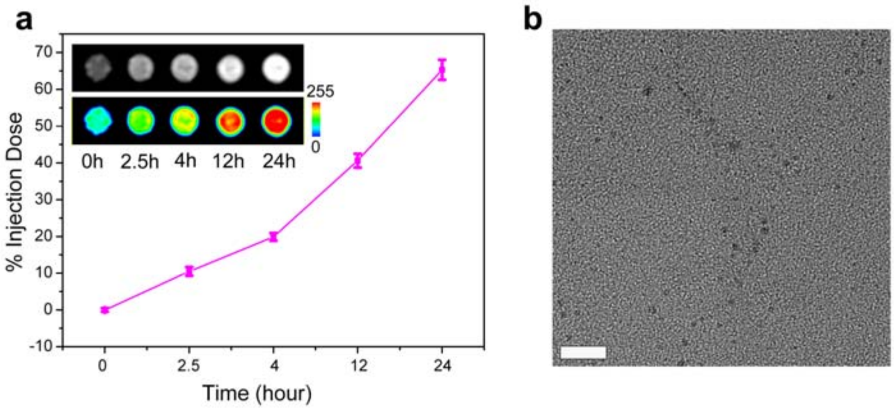

Supplementary Figure 41 | The renal clearance kinetics analysis of Fe-CPNDs. (a) Renal clearance kinetics of the Fe-CPNDs (inset: MR imaging of mouse's urine at at different time intervals). (b) TEM micrograph of mouse's urine after injection with Fe-CPNDs. Scale bar, 20 nm.

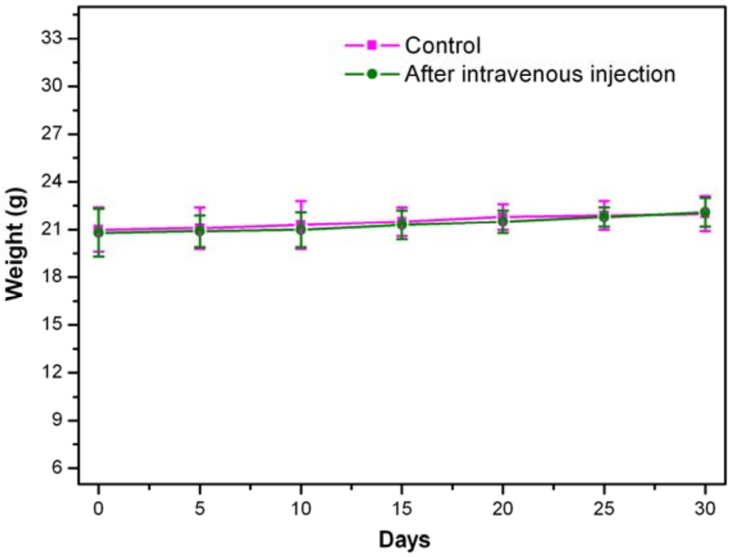

Supplementary Figure 42 | Body weight analysis. Body weight changes of the mice with and without intravenous injection of Fe-CPNDs versus time.

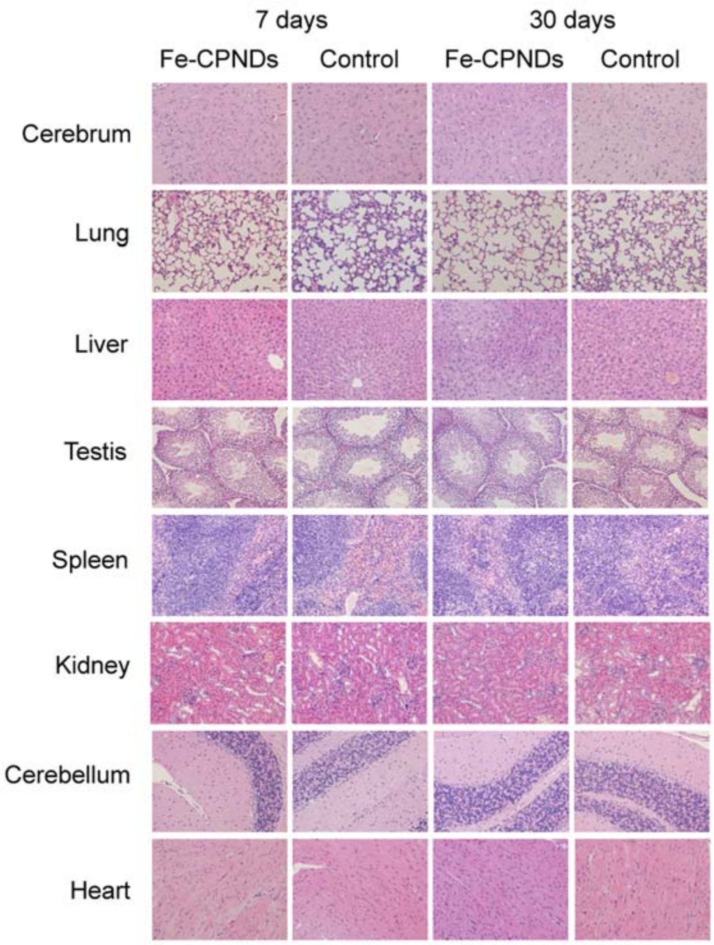

Supplementary Figure 43 | Histological analysis. Histological changes of healthy mouse with or without injection of a single dose of Fe-CPNDs after 7days and 30 days, respectively.

Bond	R (Å)	CN	σ ^{2} (Å^2)	E0(eV)	R factor
Fe-O	2.03	5.4	0.0077	4.3	0.0011
Fe-O	2.37	0.7	0.0077	4.3	

Supplementary Table 1.| Structural parameters derived from the EXAFS data analysis. Structural parameters R (Å), N, σ (Å), E0 and R factor derived from the EXAFS data analysis of Fe-CPNDs.

dispersants	hydrodynamic diameter (nm)	polydispersity index	ζ-potential (mV)
H_2O	4.2±0.25	0.132	-8.41±0.32
PBS (pH=7.4)	5.31±0.19	0.198	-1.5±0.16
TB (pH=8.5)	5.1±0.27	0.165	-5.78±0.32
0.9% NaCl solution	4.98±0.22	0.157	-4.7±0.28
DMEM (10% FBS)	5.33±0.26	0.201	-6.85±0.33

Supplementary Table 2| DLS and Zeta Potential analyses of Fe-CPNDs. DLS and Zeta Potential analysis of Fe-CPNDs solutions with different dispersants.

The HD of used PVP is about 2 nm.

Test	Units	Control (mean±sd)	Treatment (mean±sd)
Biochemistry			
AST	U/L	152.6±22.11	153.9±19.75
ALT	U/L	45.1±7.67	46.8±8.15
TP	g/L	68.9±1.26	69.8±1.53
BUN	mmol/L	6.11±0.78	6.32±1.26
CRE	μmol/L	38.8±2.13	40.2±2.57
Hematological			
WBC	$\times10^9$/L	12.32±0.99	12.65±1.98
RBC	$\times10^{12}$/L	9.65±1.25	9.59±0.78
HGB	g/L	165±1.01	166±7.25
LY	%	66.5±1.55	64.8±6.27
MCH	pg	17.10±0.89	17.30±0.62
MCHC	g/L	308.00±5.53	316.00±6.17
MCV	fL	55.40±2.56	54.80±3.26
PLT	$\times10^9$/L	780.17±39.86	770.26±51.77
PDW	fL	8.70±0.55	8.50±0.39

Supplementary Table 3| Toxicology analysis. Blood biochemical assay and hematology analysis.

Supplementary Discussion

XAS Data analysis. We used IFEFFIT software to calibrate the energy scale, to correct the background signal and to normalize the intensity.

In the Fourier transform magnitude of the EXAFS spectrum of Fe-CPNDs, three distinct peaks are observed representing the contributions of photoelectron scattering on the nearest shells of neighbors around the Fe atom. A strong peak in the R range between 1.5 and 2.2 Å can be attributed to photoelectron backscattering on the nearest neighbors around Fe. The second peak between 2.2 and 2.5 Å in the R range represents the contributions from more distant Fe coordination shells.

Three variable parameters for each shell of neighbors are introduced in the model: shell coordination number (N), distance (R), and Debye-Waller factor (σ^2). Avery good agreement between the model and experimental spectrum is found using the k range of 2.5-10.8 $Å^{-1}$, and the R range from 0.8 to 2.6 Å. The list of best fit parameters is given in Supplementary Table 1.

Supplementary Methods

XAFS Data Collection. The X-ray absorption data at the Fe *K*-edge of the samples were recorded at room temperature in transmission mode using ion chambers or in the fluorescent mode with silicon drift fluorescence detector at beam line BL14W1 of the Shanghai Synchrotron Radiation Facility (SSRF), China. The station was operated with a Si(111) double crystal monochromator. During the measurement, the synchrotron was operated at energy of 3.5GeV with a current between 150-210 mA.

The photon energy was calibrated with the first inflection point of Fe K-edge in Fe metal foil.

The Fe K-edge XANES analysis is used in this work to determine the average Fe valence state in the Fe-CPNDs. The edge energy of the Fe K-edge in the NPs sample is close to the Fe^{3+} compounds, confirming that the iron in Fe-CPNDs is predominantly ferric ion.

FTIR characterization at different temperatures. The infrared spectra were conducted with a Bruker Vertex 70 Fourier transform infrared spectrometer (FT-IR) increasing from room temperature to 188 °C at a rate of 10 ^{o}C min^{-1} and maintained for 1 h. The intensity of IR band at 3440 cm^{-1} is decreased with increasing the temperature, indicating that coordinated water molecules have been released from the Fe-CPNDs. The IR intensity change of Fe-CPNDs incubated at pH 5.0 is stronger than that of Fe-CPNDs incubated at pH 7.4, suggesting that Fe-CPNDs were degraded[1].

FTIR characterization of CO adsorbed. In situ FTIR spectra were taken with a Nicolet 6700 spectrometer at a resolution of 4 cm^{-1}. The fine powder of the Fe-CPNDs was mounted in a ceramic crucible. The sample was pretreated in Ar at 25 °C for 20 min and was then exposed to a stream of 1 vol % CO/Ar. The absorption bands centered at *ca.* 2175 cm^{-1} were assigned to CO adsorbed on Fe^{3+} sites[2].

Degradation characterization. The degradation of Fe-CPNDs was studied by dissolving 15 mg of the Fe-CPNDs in 2 mL buffer solution and introduced into a dialysis bag (MWCO=8000). The bag was then immersed into a container with 10 mL

of buffer solution at the same pH value as that inside the bag. The outer phase of the buffer solution was oscillated at 37 °C. At selected time intervals, 1 mL of the external buffer was collected for HPLC measurement and an equal volume of fresh buffer was replenished. The amount of GA was determined by HPLC (SHIMADZU CBM-10A VPPLUS) with C18 column. Methanol-0.2% Formic acid solution (15:85) as the mobile phase, flow rate 0.7 ml/min, detection wavelength was 265 nm and column temperature was 25 °C. GA solutions with concentrations of 0.01, 0.1, 1, 10 and 100 μg mL^{-1} were used as standards. The degradation rate of Fe-CPNDs were calculated by following equation, %Degradation $=M_{GA\ released}/M_{GA\ total}$; %Degradation means the degradation rate of Fe-CPNDs, $M_{GA\ released}$ means the amount of GA released from Fe-CPNDs with various pH values, $M_{GA\ total}$ means the total amount of GA in Fe-CPNDs.

Synthesis of PVP-Fe. PVP (66 mg) was dissolved in 8.8 mL water at room temperature under vigorous stirring. And then, $FeCl_3$ aqueous solution (0.2 mL, 100 mg mL^{-1}) was added into the aqueous PVP solution. After 1 h incubation, the resulting complex were dialyzed (MWCO = 25000) against deionized water for 24 h.

Synthesis of GA-Fe, PVP-GA and PVP-Fe. *Synthesis of GA-Fe:* $FeCl_3$ (20 mg) was added into a water solution (9 mL). After 1 h incubation, GA aqueous solution (1 mL, 10 mg mL^{-1}) was introduced into the above reaction mixture and stirred overnight. The resulting nanodots were dialyzed (MWCO = 25000) against deionized water for

24 h. *Synthesis of PVP-GA and PVP-Fe:* PVP (66 mg) was dissolved in water solution (9 mL) at room temperature under vigorous stirring. After 1 h incubation, $FeCl_3$ aqueous solution (1 mL, 20 mg mL^{-1}) or GA aqueous solution (1 mL, 10 mg mL^{-1}) was added into the aqueous PVP solution and stirred overnight. The resulting nanodots were dialyzed (MWCO = 25000) against deionized water for 24 h.

Large-scale Synthesis of Fe-CPNDs. PVP (3.63 g) was dissolved in water (428 mL) at room temperature under vigorous stirring. And then, $FeCl_3$ aqueous solution (12 mL, 100 mg mL^{-1}) was added into the aqueous PVP solution. After 1 h incubation, GA aqueous solution (60 mL, 10 mg mL^{-1}) was introduced into the above reaction mixture and stirred overnight. The resulting nanodots were dialyzed (MWCO = 25000) against deionized water for 24 h.

***In vivo* Biodistribution analysis.** The nude mice bearing colorectal tumor were used to examine the in vivo biodistribution of the Fe-CPNDs. The mice were first anesthetized by an intraperitoneal injection of 10 % chloralhydrate (80 μL). A NaCl solution (0.9 wt%) containing Fe-CPNDs was then intravenous (2 mg kg^{-1}) or intratumor (0.25 mg kg^{-1}) injected into the mice. The mice were euthanized, and the heart, liver, spleen, lung, kidney, bladder and tumor were extracted and weighted at different time points (1.5, 2.5, and 24 h post injection for the intravenous injection case; 15 min and 45 min post injection for the intratumor injection case). The tissues were first rinsed in PBS and then lyophilized for 24 h to obtain dry tissue. The dry

tissue was then digested with 3 mL of concentrated nitric acid for 24 h. After digestion, the samples were filtered with 0.450 μm syringe filters and dissolved in deionized water (Millipore, USA) to bring the final volume to 10 mL. The organs of untreated nude mouse were employed as control samples. The relative amounts (%) of Fe in different organs (% ID g^{-1}) were calculated by following equation, % ID $g^{-1}=(M_{Fe\ treated}-M_{Fe\ control})/(M_{Fe\ total}\times Weight_{organ})$; $M_{Fe\ treated}$ means the amount of Fe found in organ of Fe-CPNDs treated mouse, $M_{Fe\ control}$ means the amount of Fe found in organ of untreated mouse, and $M_{Fe\ total}$ means the total amount of Fe injected, respectively.

***In vivo* infrared thermal images analysis.** An IR thermal camera (FLIR i3) was used to monitor the real-time temperature change of the tumor tissue during 808 nm NIR laser irradiation. Upon 808 nm NIR laser irradiation, Fe-CPNDs treated mouse showed localized heating in the tumor site, where the temperatures were greatly increased after 6 min irradiation. The tumor temperatures of 0.9% NaCl solution treated mouse exhibited no significant increase of temperature at tumor site during 6 min of laser irradiation. It confirmed the high accumulation of Fe-CPNDs inside the tumor tissue and its ability to induce hyperthermia by converting NIR light energy to heat *in vivo*.

Pharmacokinetics analysis. To determine the blood circulation time of the Fe-CPNDs, the bloods were collected and mixed with a saline flush containing 100

unit mL^{-1} heparin at 5, 10, 20, 40, 60, 120, 300, 720, and 1440 min post-injection. The bloods of untreated mouse were collected under same conditions and employed as control samples. Fe content in blood was measured by ICP-MS. The relative amounts (%) of Fe in bloods of treated mice were calculated by following equation, % ID g^{-1}=($M_{Fe\ treated}$-$M_{Fe\ control}$)/($M_{Fe\ total}$×$Weight_{blood}$); $M_{Fe\ treated}$ means the amount of Fe found in blood of Fe-CPNDs treated mouse, $M_{Fe\ control}$ means the amount of Fe found in blood of untreated mouse, and $M_{Fe\ total}$ means the total amount of Fe injected, respectively. Blood half-life was calculated with a two exponential fit using a two-compartment mode.

Renal clearance kinetics analysis. To quantitative analysis of excreted Fe-CPNDs, the mice were first anesthetized by an intraperitoneal injection of 10 % chloralhydrate (100 μL). A NaCl solution (0.9 wt%) containing Fe-CPNDs (2 mg kg^{-1}) was then intravenous injected into the mice. After that, the mice were placed in the standard metabolic cages and the urines were collected with special precaution to avoid fecal contamination at 0, 2.5, 4, 12 and 24 h post-injection, and imaged by MRI after concentration. The mice were released from sedation after two hours. The urine of untreated was collected under same conditions and employed as control samples. Fe content in urine was measured by ICP-MS. The relative amounts (%) of Fe in urine of treated mice were calculated by following equation, %Injection Dose=($M_{Fe\ treated}$-$M_{Fe\ control}$)/$M_{Fe\ total}$; $M_{Fe\ treated}$ means the amount of Fe found in urine of Fe-CPNDs treated mouse, $M_{Fe\ control}$ means the amount of Fe found in urine of untreated mouse, and M_{Fe}

total means the total amount of Fe injected, respectively. For TEM experiment, the urine was dialyzed (MWCO = 25000) against deionized water for 24 h before TEM measurement.

Supplementary References

1. Nag, J., Pal, S. & Sinha, C. Synthesis and characterization of cobalt(II), nickel(II), copper(II), palladium(II) and dioxouranium(VI) complexes of the antipyrine Schiff base of 3-formylsalicylic acid. *Transit. Metal chem.* **30**, 523-526 (2005).
2. Lemire, C., Meyer, R., Henrich, V.E., Shaikhutdinov, S. & Freund, H.J. The surface structure of Fe_3O_4(111) films as studied by CO adsorption. *Surf. Sci.* **572**, 103-114 (2004).
